# USP39 promotes hepatocellular carcinogenesis through regulating alternative splicing in cooperation with SRSF6/HNRNPC

**DOI:** 10.1038/s41419-023-06210-3

**Published:** 2023-10-11

**Authors:** Jingyi Zheng, Shasha Wu, Mao Tang, Shaoyan Xi, Yanchen Wang, Jun Ren, Hao Luo, Pengchao Hu, Liangzhan Sun, Yuyang Du, Hui Yang, Fenfen Wang, Han Gao, Ziwei Dai, Xijun Ou, Yan Li

**Affiliations:** 1 Department of Biology, School of Life Sciences, Southern University of Science and Technology, Shenzhen, China; 2 Department of Pathology, Sun Yat-Sen University Cancer Center, Guangzhou, China; 3 State Key Laboratory of Oncology in South China and Collaborative Innovation Center for Cancer Medicine, Sun Yat-sen University Cancer Center, Guangzhou, China

**Keywords:** Liver cancer, Alternative splicing

## Abstract

Abnormal alternative splicing (AS) caused by alterations in spliceosomal factors is implicated in cancers. Standard models posit that splice site selection is mainly determined by early spliceosomal U1 and U2 snRNPs. Whether and how other mid/late-acting spliceosome components such as USP39 modulate tumorigenic splice site choice remains largely elusive. We observed that hepatocyte-specific overexpression of USP39 promoted hepatocarcinogenesis and potently regulated splice site selection in transgenic mice. In human liver cancer cells, USP39 promoted tumor proliferation in a spliceosome-dependent manner. USP39 depletion deregulated hundreds of AS events, including the oncogenic splice-switching of KANK2. Mechanistically, we developed a novel RBP-motif enrichment analysis and found that USP39 modulated exon inclusion/exclusion by interacting with SRSF6/HNRNPC in both humans and mice. Our data represented a paradigm for the control of splice site selection by mid/late-acting spliceosome proteins and their interacting RBPs. USP39 and possibly other mid/late-acting spliceosome proteins may represent potential prognostic biomarkers and targets for cancer therapy.

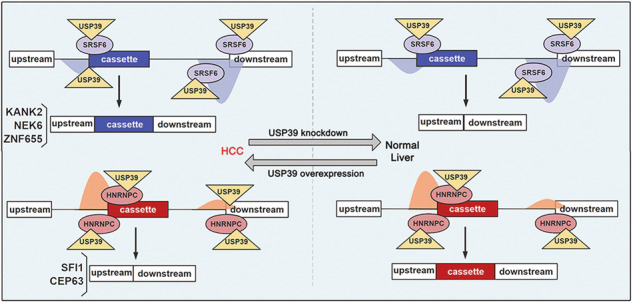

## Introduction

Alternative splicing (AS) is an important mechanism of RNA processing that generates different mRNA/protein isoforms from a single mRNA transcript, thereby enlarging the diversity and complexity of the transcriptome and proteome. Pre-mRNA splicing is carried out by the spliceosome, a megadalton complex comprising five small nuclear ribonucleoprotein particles (U1, U2, U4/5/6 snRNP) and over 100 core set proteins. The key to AS regulation lies in the selection of splice sites, which is believed to be mainly determined by U1 and U2 snRNPs, due to their direct recognition of 5’/3’ splice sites. Additional interactions that regulate splice site selection are mediated by RBPs (e.g., SR and hnRNP protein families), which recognize auxiliary sequences in the pre-mRNA to promote or inhibit complex A assembly. Subsequent binding of preassembled U4/5/6 tri-snRNP forms complex B, which undergoes a series of conformational changes to form complexes Bact and C, and concomitantly carries out the two trans-esterification reactions to generate splicing intermediates and products [1].

Over the past decade, rapid developments in high-throughput technologies have revealed broad alterations in splicing in various cancers [2–4]. Genetic alteration and/or abnormal expression of spliceosomal components have been frequently detected and contribute to the abnormal splicing patterns in tumors. These findings indicate that cancer-related isoforms and various splicing regulatory factors can be used as potential targets in cancer therapy, leading to a new treatment strategy called spliceosome-targeted therapies [5–7]. U1 and U2 snRNP components have been intensively studied and pharmacologically targeted because of their direct influence on splice site recognition and frequent mutation in hematological tumors [8, 9]. Small molecules targeting these components, such as SF3B inhibitors, cause severe toxic side effects due to their general regulation of splicing efficiency, although they are effective in various cancers [10]. On the other hand, targeting non-spliceosomal regulators such as RBM39 is less toxic, however, its efficacy in cancer patients is limited. The current dilemma prompted researchers to turn to other mid- or late-acting spliceosome components for solution. This may be especially true for cancer types like hepatocellular carcinoma (HCC) because extensive abnormal expression of spliceosomal components rather than mutation of U1/U2 snRNP components is frequently detected in these cancers [11].

Several key issues need to be addressed whether mid- and late-acting spliceosome components can be used as therapeutic targets. Despite extensive deregulation of spliceosomal gene expression, in vivo, models and data are needed to determine whether these proteins are drivers or mere passengers in cancer pathogenesis. Considering standard models of AS regulation at the early steps of spliceosome assembly, we wonder whether and how mid- and late-acting spliceosome components modulate splice site choice, rather than only affect the efficiency of splicing. Take Ubiquitin-specific protease 39 (USP39), a component of U4/U6. U5 tri-snRNP, as an example. USP39 is an ortholog of yeast Sad1 and was first identified to facilitate U4/U6.U5 tri-snRNP assembly and complex B formation and conversion to Bact [12, 13]. It is also defined as a member of the deubiquitylation family but is considered to be devoid of deubiquitinating activity due to the absence of three crucial catalytic residues. The aberrant expression of USP39 has been implicated in the tumorigenesis of various malignancies [14, 15]. Overexpression of USP39 facilitates constitutive splicing of individual pre-mRNAs in different cancers [16–20]. In addition, USP39 regulates SP1 and ZEB1 protein stability via deubiquitylation [21, 22]. However, as a mid/late-acting spliceosome protein, the roles and targets of USP39 in alternative splicing regulation remain unclear. Little is known about the mechanism underlying USP39-mediated splice site selection in malignancies.

Herein, we found that the extensive upregulation of spliceosome components is a molecular feature of HCC. Using a conditional *Usp39* overexpression mouse model, we demonstrated that USP39 was a driver of liver cancer as well as a potent regulator of AS. The splicing landscape regulated by USP39 has been comprehensively characterized in human HCC cells, and KANK2 is one of the functionally important AS targets. Mechanistically, USP39 modulates alternative splice site choice by interacting with SRSF6 and HNRNPC in a conserved manner between humans and mice. USP39 serves as a paradigm of the mid- or late-acting spliceosome components that display potent regulatory properties of AS. These spliceosomal proteins represent potential targets of spliceosome-targeted therapies.

## Results

### USP39-overexpression associates with HCC pathogenesis and aberrant cell cycle signaling

**Fig. 1:**
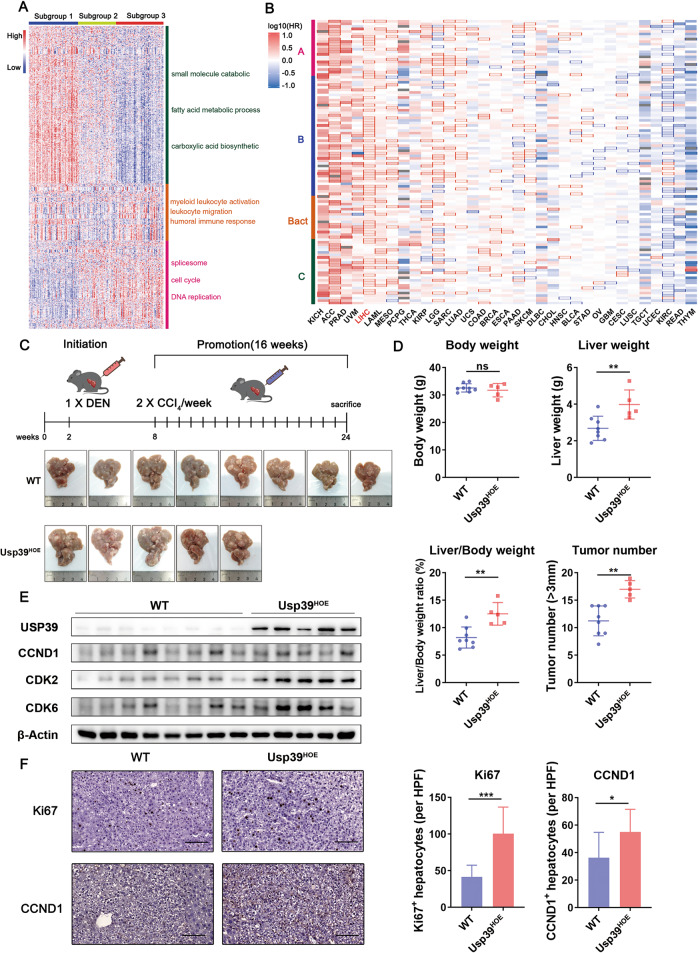
Overexpression of spliceosome components associates with HCC pathogenesis and conditional USP39 overexpression enhances in vivo hepatocarcinogenesis. **A** Proteomic subgroups were identified based on differentially expressed proteins between tumor and non-tumor tissues from the CPTAC HCC cohort. Each column represents a patient sample and rows indicate proteins. The color of each cell shows the *Z*-score (log2 of relative abundance scaled by protein SD) of the protein in that sample. **B** HR heatmap representing associations between spliceosomal gene expression and OS across 33 different cancer types profiled using TCGA. The HR for the high expression group versus the low expression group (median expression value as a cut-off) was calculated. The heatmap is colored based on the log10 HR. A square with a bold border represents a *p*-value < 0.05 in the survival analysis. Each column represents a cancer type, and rows indicate genes arranged in the order of spliceosome assembly (A: complex A, B: complex B, Bact: complex B catalytic active, C: complex C). **C** Schematic summary of the DEN/CCl4-induced hepatocarcinogenesis model established in *Usp*39^HOE^ and control mice (WT). Images of tumor-bearing livers are shown. **D** Body weight, liver weight, liver/body weight ratios, and tumor numbers of WT and *Usp*39^HOE^ mice were measured. ns: not significant. **E** USP39 and cell cycle-related proteins including CCND1, CDK2, and CDK6 were detected with WB. **F** Immunohistochemistry was performed to examine the expression of Ki67 and CCND1 in WT and *Usp*39^HOE^ mice. The IHC-positive hepatocytes were then counted. Three high-power fields (HPF, ×800 magnification) were obtained from each mouse (*n* = 3). Scale bar, 100 μm. Mean ± SD. *P* values were determined using an unpaired Student’s *t*-test. ∗*P* < 0.05, ∗∗*P* < 0.01, ∗∗∗*P* < 0.001. (Experimental group, *n* = 5; control group, *n* = 8).

Recent high-throughput technologies have revealed the molecular landscape of HCC. Using a proteomic dataset from CPTAC, we performed unsupervised clustering based on proteins differentially expressed between tumor (T) and non-tumor (NT) livers. Consistent with a previous study [23], three subgroups were identified among the 159 tumors. Subgroup 1 was enriched with metabolism-related proteins and subgroup 2 was characterized by immune deregulation. Subgroup 3, with the worst prognosis as reported by Gao et al. [23], harbored the highest cell renewal pathways, such as cell cycle, DNA replication, and spliceosome (Fig. 1A, Table S1), suggesting that spliceosome proteins may be drivers of HCC subgroup.

The spliceosome is a megadalton complex comprising over 100 proteins [2]. We studied the effect of spliceosomal gene expression on overall survival (OS) using TCGA data pan-cancer analysis. The hazard ratio (HR) heatmap results showed that spliceosomal gene expression was a robust biomarker for prognosis in various cancers, including liver hepatocellular carcinoma (LIHC) (Fig. 1B, Table S2). Among these, the expression of *USP39*, a core tri-snRNP component, was upregulated in paired and unpaired HCC tissues in both TCGA and GEO cohorts (Fig. S1A, B). Elevated USP39 expression in tumor tissues at both the protein and mRNA levels has also been demonstrated in previous studies [22, 24, 25]. High *USP39* expression was associated with adverse clinicopathological features and poor prognosis (Fig. S1C, D). Consistent with the proteome profile in Fig. 1A, cell cycle, and DNA replication pathways were enriched in tumors with high *USP39* mRNA expression via gene set enrichment analysis (GSEA) (Fig. S1E). These results highlighted the link between USP39 overexpression and HCC pathogenesis, together with changes in cell cycle signaling.

### Conditional USP39 overexpression enhances in vivo hepatocarcinogenesis

The clinical data analysis revealed extensive deregulation of spliceosomal genes during HCC development. Whether USP39 serves as a functionally important driver or just a passenger remains to be determined. To establish the causal relationship between USP39 upregulation and hepatocarcinogenesis, hepatocyte-specific *Usp39* overexpression mice (*Usp39*^HOE^) were generated by crossing *Rosa26-stopflox/flox-Usp39* mice with *Albumin-Cre* mice. In the DEN/CCl4-induced hepatocarcinogenesis model, we observed more severe liver tumorigenesis in *Usp39*^HOE^ mice than in wild-type (WT) mice (Fig. 1C, D). Along with these macroscopic observations, the molecular levels of CCND1, CDK2, CDK6, and Ki67, proteins that promote and mark cell cycle progression, were also upregulated (Fig. 1E, F).

### USP39 extensively modulates alternative splicing through potential interactions with RBPs

**Fig. 2:**
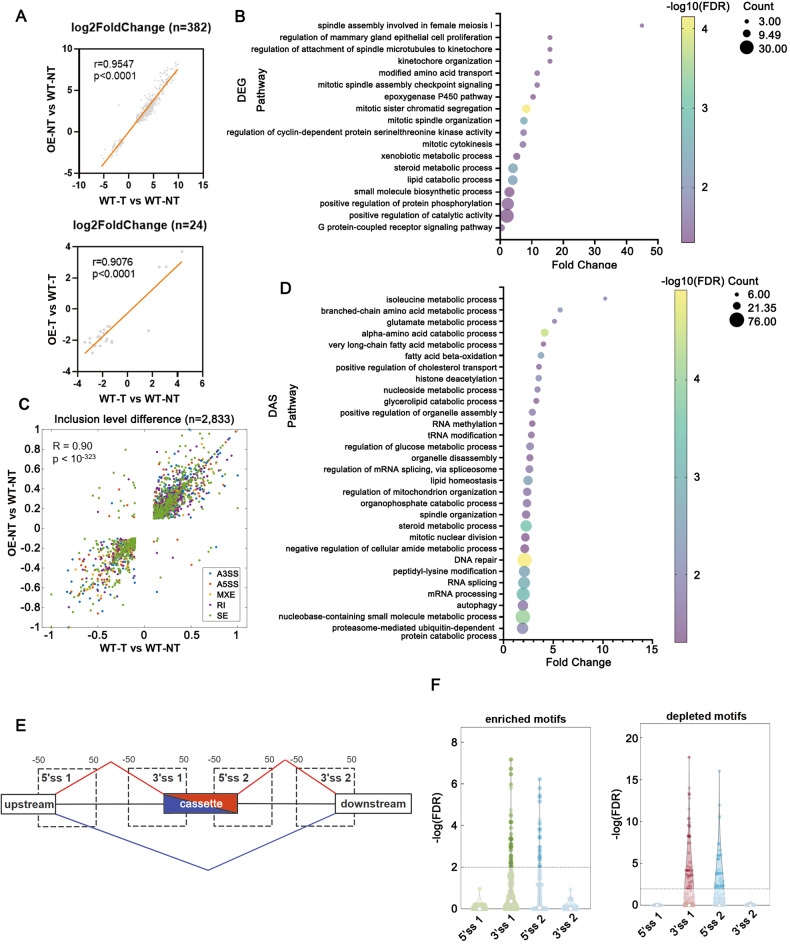
USP39 extensively modulates alternative splicing in mice through potential interactions with RBPs. **A** The overlapping DEGs in OE-NT vs. WT-NT and WT-T vs. WT-NT datasets (up, *n* = 382) and OE-T vs. WT-T and WT-T vs. WT-NT datasets (down, *n* = 24) were positively correlated. (Pearson Correlation test was performed.) **B** The GO biological process (BP) enrichment analysis of the overlapping DEGs in OE-NT vs. WT-NT and WT-T vs. WT-NT datasets. **C** The overlapping DAS events (*n* = 2833) were positively correlated in the OE-NT vs. WT-NT and WT-T vs. WT-NT datasets. **D** The GO biological process (BP) enrichment analysis of the overlapping DAS events in OE-NT vs. WT-NT and WT-T vs. WT-NT datasets. **E** and **F** RBP motif enrichment analyses within the four regions (5’ss1, 3’ss1, 5’ss2, 3’ss2) around the regulated cassette exons were compared with those around the background cassette exons. The start and end of each region were labeled by a nucleotide distance to the splice site. **E** Enriched motifs had a predominant distribution within the indicated regions around the regulated cassette exons compared with the background exons, while depleted motifs had less distribution around the regulated exons. The histogram described the enriched (left) or depleted (right) motifs in the indicated regions (**F**).

To further examine how USP39 promotes tumorigenesis in vivo, tumor and para-tumor tissues collected from *Usp39*^HOE^ and WT mice were analyzed by transcriptome sequencing. Tumorigenesis-related differentially expressed genes (DEGs) and differentially alternative splicing (DAS) events were identified by comparing wild-type tumors with wild-type non-tumors (WT-T vs. WT-NT) (Fig. S2). USP39-regulated events were determined by comparing *Usp39*-overexpressing non-tumors/tumors with wild-type non-tumors/tumors (OE-NT vs. WT-NT and OE-T vs. WT-T) (Fig. S2). Compared to DEGs regulated by USP39 in tumors, more DEGs regulated by USP39 in non-tumors overlapped and had positive correlation with tumorigenesis-related DEGs, suggesting that USP39 mainly played a tumorigenic role during the early stage of tumor formation (Fig. 2A). Consistently, DAS events regulated by USP39 in the early stage (OE-NT vs. WT-NT) overlapped and had remarkably high positive correlation coefficient with tumorigenesis-related DAS events (WT-T vs. WT-NT), strongly suggesting their relevance for neoplastic transformation (Fig. 2C). These overlapping DEGs and DAS genes were enriched in mitotic cell cycle control, cell proliferation, DNA repair and related metabolic processes (Fig. 2B, D). We wondered how this mid/late-acting spliceosome component potently influenced splice site choices. Unlike splicing factors, such as SR and hnRNP proteins, USP39 itself does not possess an RNA-binding domain. We speculated that the specificity of USP39-regulated AS relied on the interplay of USP39 with RBPs, which facilitated the interaction of the spliceosome with corresponding RBP-binding motifs in pre-mRNAs. We performed RBP motif enrichment analysis to determine whether the distribution of the RBP-binding motifs differed between USP39-regulated cassette exons and background exons. The overlapping skip exon (SE) events in Fig. 2C (921 events) were defined as USP39-regulated cassette exons while 150,000 exons were randomly selected from the GRCm39 reference genome as background exons. Consensus motifs of RBPs were downloaded from ATtRACT database and their occurrences around the 5’/3’ splice sites were analyzed (Fig. 2E). Dozens of RBP motifs were identified to be significantly enriched or depleted within the cassette exons over background exons, but not in the upstream/downstream exons, suggesting that given RBPs have an important role in USP39-regulated AS (Fig. 2F). In contrast to standard models of AS regulation at an early stage of spliceosome assembly, these results provided in vivo evidence that the mid/late-acting spliceosomal component USP39 can potently affect splice site selection, possibly via interaction with RBPs.

### USP39 exerts its pro-proliferative effect in a spliceosome-dependent manner

**Fig. 3:**
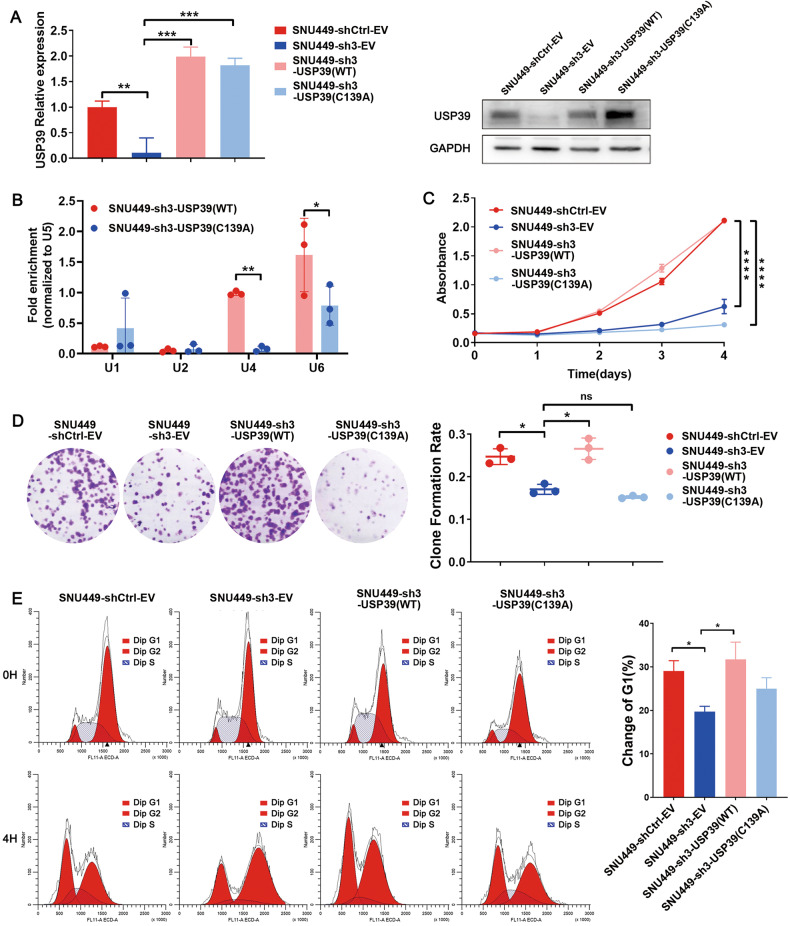
USP39 exerts its pro-proliferative effect in a spliceosome-dependent manner. **A** Flag-USP39 (WT) and Flag-USP39 (C139A) were introduced to USP39-deficiency (sh3) *SNU-449* cells and verified with qRT-PCR and WB assays. **B** RIP experiments were performed using antibodies against Flag in the indicated cells. The levels of U1, U2, U4, and U6 snRNA in immunoprecipitated RNA were detected using qRT-PCR and normalized to the levels of U5 snRNA in each sample (*n* = 3). **C** CCK8 assay showed that only wild-type USP39, but not C139A mutant, could rescue the inhibition effect of USP39 deficiency on cell proliferation. **D** Representative images and quantification of foci formation induced by the indicated cells (*n* = 3). **E** Cells were synchronized at the G2/M boundary after being treated with thymidine and nocodazole. Following the release, the cells arrested in G2/M (0 h) would reenter the G1 phase (4 h). Cell cycle profiles showed changes in G1 cell populations at 4 h post-release. C139A mutant failed to rescue cell cycle arrest caused by USP39 knockdown (*n* = 3). Mean ± SD. *P* values by paired (**B**) or unpaired Student’s *t*-test (**A**, **C**–**E**). ∗*P* < 0.05, ∗∗*P* < 0.01, ∗∗∗*P* < 0.001, ∗∗∗∗*P* < 0.0001. ns: not significant.

Consistent with its phenotype in a mouse model, USP39 displayed oncogenic functions in human HCC cell lines. *PLC-8024* and *SNU-449* cells were chosen for further experiments, representing distinct human HCC cells with different USP39 expression levels. ShRNA-mediated USP39 silencing reduced cell viability, foci formation frequencies, colony formation in soft agar, and cell cycle progression, whereas USP39 overexpression increased these capabilities (Figs. S3–S4). It has been reported that USP39 acted as a deubiquitinase as well as a spliceosome component. We wondered whether USP39 promoted HCC cell proliferation by regulating spliceosome assembly. A previous study showed that a C63A mutation in Sad1, the homolog of USP39 in yeast, disrupts its interaction with Snu114 and Snu66, thereby impairing tri-snRNP formation [26]. As the core spliceosome component USP39 is highly conserved across species, a USP39-C139A mutant, which is equivalent to the Sad1-C63A mutant, was introduced into USP39-knockdown HCC cells (Figs. 3A, S5–7A). This point mutation was validated to interrupt tri-snRNP assembly in human HCC cells. RNA immunoprecipitation (RIP) results showed that the decreased U4 and U6 snRNAs were co-purified with the C139A mutant, which indicated specific defects in tri-snRNP biogenesis (Figs. 3B, S5–7B). Unlike wild-type USP39, which rescued the inhibitory effect of USP39 deficiency on cell proliferation and cell cycle progression, the C139A mutant could not reverse the suppressive effects (Figs. 3C–E, S5–7C–E). These results suggested that USP39 exerts its pro-proliferative effects mainly in a spliceosome-dependent manner.

### USP39-knockdown reduces global splicing efficiency and selectively regulates AS in human HCC

**Fig. 4:**
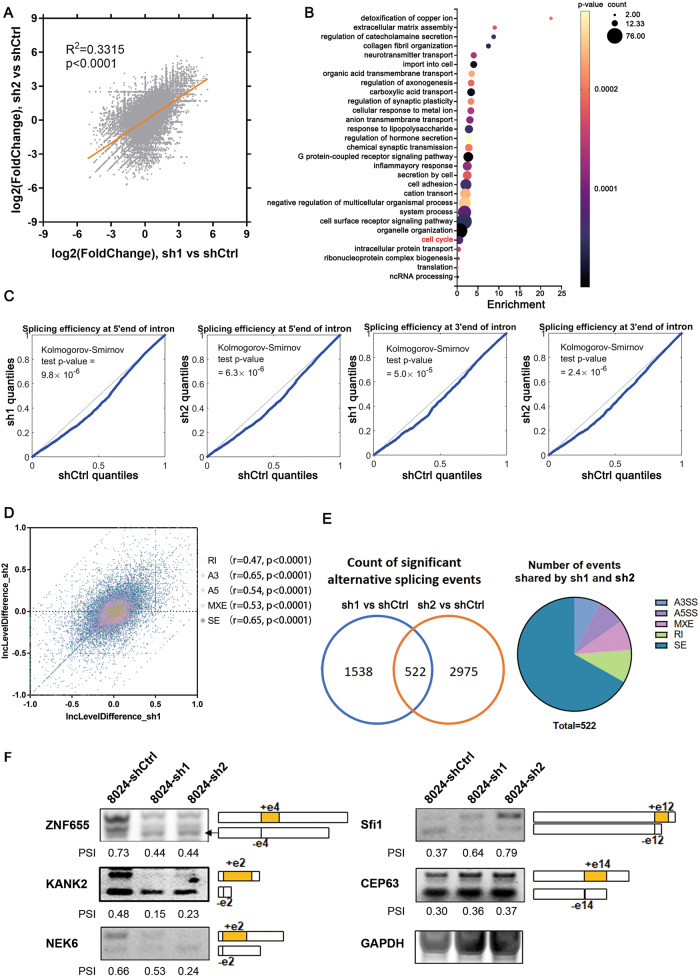
USP39 knockdown reduces global splicing efficiency and selectively regulates alternative splicing. **A** *PLC-8024* USP39-knockdown (sh1, sh2) and control (shCtrl) cells were collected for transcriptome sequencing. Sh1 showed a similar DEG pattern to sh2. **B** The GO biological process (BP) enrichment analysis of the overlapping DEGs. **C** Global splicing efficiency analysis at 5′ and 3′ splice sites in *PLC-8024* cells with and without USP39 depletion. **D** DAS events were highly correlated in sh1 vs. shCtrl and sh2 vs. shCtrl datasets. **E** Venn diagram displayed the DAS events in sh1 vs. shCtrl and sh2 vs. shCtrl datasets after the intersection (left). Pie chart depicting the proportions of different types of overlapping DAS events (right). A5SS alternative 5’ splice site, A3SS alternative 3’ splice site, MXE mutually exclusive exons, RI retained intron, SE skipped exon. **F** Three events with down-regulated PSI values (left) and two targets with up-regulated PSI values (right) in USP39 depletion *PLC-8024* cells were validated with RT-PCR and agarose gel electrophoresis. The percentages of cassette exon inclusion over the total transcripts were presented using PSI.

To verify whether USP39 also regulates human AS, *PLC-8024* USP39-knockdown and control cells were collected for transcriptome sequencing. As shown in Fig. 4A, two independent shRNAs (sh1 and sh2) targeting USP39 generated highly correlated DEG patterns, confirming the reliability of the RNA-seq data. Gene ontology (GO) enrichment analysis of these overlapping DEGs showed that USP39 depletion significantly affected pathways such as cell adhesion, translation, and cell cycle (Fig. 4B). As USP39 is required for spliceosome complex B assembly and catalytic activation, we investigated whether *USP39* expression affects pre-mRNA splicing. We calculated splicing efficiency using RNA-seq data and found a widespread impairment in pre-mRNA splicing upon the loss of USP39. Both 5’ and 3’ splice site efficiencies were significantly reduced (Fig. 4C), suggesting that USP39 acts as a global regulator of splicing efficiency. The differences in AS events were further analyzed, and these two USP39 deficient cells shared strongly correlated DAS patterns, which ensured data reliability (Fig. 4D). 522 overlapping DAS events (Δ percent spliced in, ΔPSI > 0.1, and adjusted *P*-value < 0.05) were detected in sh1 and sh2 knockdown cells. Among these 522 events, 349 were skipped exon (SE) events with either increased or decreased PSI values (Fig. 4E). Five target genes that have been reported to influence cell viability or cell cycle transition were selected and verified by RT-PCR (Fig. 4F).

### USP39 promotes tumorigenesis partially through a splicing switch of KANK2

**Fig. 5:**
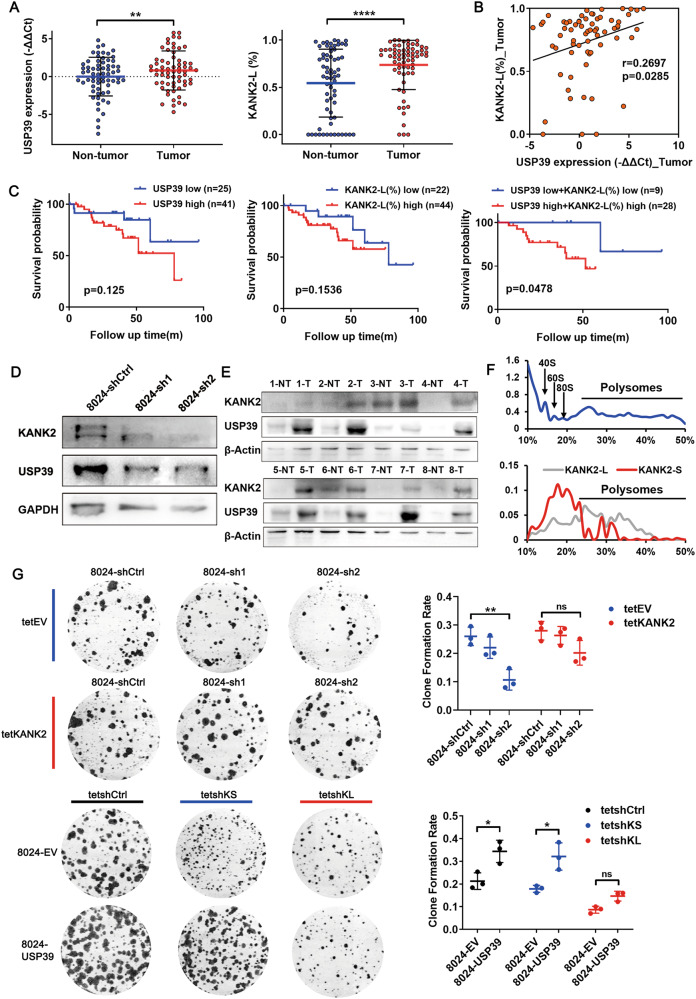
USP39 exerts its malignant effects partially through an isoform switch of KANK2-S to KANK2-L. **A** The mRNA level of USP39 and the expression proportion of KANK2-L isoform were detected in our in-house HCC cohort (*n* = 66). **B** The expression proportion of KANK2-L isoform was positively correlated with USP39 mRNA expression in tumor tissues (Pearson Correlation test). **C** Kaplan–Meier OS curves of in-house HCC patients based on USP39 expression (high vs. low, defined as tumor/non-tumor > 1) and KANK2-L percentage (high vs. low, defined as tumor(%)/non-tumor(%) > 1). **D** WB assay revealed KANK2 protein reduction in USP39 knockdown cells. **E** WB analysis of adjacent non-tumor (NT) and tumor (T) tissue lysates from HCC patients with USP39 and KANK2 antibodies. β-Actin was used as a loading control. **F** Polyribosome profile analysis revealed that KANK2-L isoform enhanced the translation of KANK2 protein. **G** Tet-KANK2 was introduced to USP39-deficient *PLC-8024* cells (up). Two Tet-On shRNAs targeting KANK2-L (tetshKL) and KANK2-S (tetshKS) were introduced into USP39-overexpressing *PLC-8024* cells (down). Foci formation assay was performed to assess the growth of the indicated cells. Representative images and statistical results are shown (*n* = 3). Mean ± SD. *P* values by paired (**A**) or unpaired (**G**) Student’s *t*-test. *P < 0.05, **P < 0.01, ns: not significant.

Among the USP39-affected DAS events, we focused on the KN motif and ankyrin repeat domains 2 (KANK2) gene, as it displayed the most significant alteration in splicing patterns during our preliminary screening of clinical HCC samples (Fig. S8). KANK2 is a focal adhesion protein that regulates cell adhesion, migration, apoptosis, and proliferation [27–29]. Knockdown of USP39 resulted in a decrease in the long isoform of KANK2 (KANK2-L, containing exon 2) relative to its short isoform (KANK2-S, lacking exon 2) (Fig. 4F). Based on these in vitro findings, we first examined the clinical significance of these two KANK2 isoforms by comparing the proportion of KANK2-L mRNA in 66 human HCC and adjacent non-tumor tissues. Both the expression of USP39 and the inclusion of KANK2 exon 2 were significantly increased in tumor samples compared to non-tumor samples in this in-house HCC cohort (Fig. 5A). A positive correlation was observed between *USP39* mRNA expression and the percentage of KANK2-L (Fig. 5B). Patients with either higher levels of *USP39* expression or a higher proportion of the KANK2-L isoform had poorer prognosis, although this was not statistically significant due to the limited number of cases. Notably, patients with relatively high *USP39* expression and a high proportion of KANK2-L showed significantly worse OS than those with relatively low *USP39* expression and a low proportion of KANK2-L (Fig. 5C).

We examined the molecular functions of the two KANK2 isoforms. Exon 2 is located in the 5’-untranslated region (UTR) of KANK2 and does not affect the protein sequence. A marked reduction in KANK2 protein was observed in accordance with the isoform switch in USP39 knockdown cells (Fig. 5D). Furthermore, a positive correlation was observed between USP39 and KANK2 protein expression in HCC clinical samples (Fig. 5E). Therefore, we hypothesized that the KANK2-L isoform enhances the translation of the KANK2 protein compared to KANK2-S. Supporting this, polyribosome profile analysis revealed that a higher proportion of KANK2-L was associated with heavy polyribosomes, whereas KANK2-S was largely associated with light polyribosomes (Fig. 5F). Mass spectrometry analysis of RNA pulldown samples showed that KANK2-L mRNA bound more translation-related proteins than KANK2-S mRNA, indicating a higher translation efficiency for KANK2-L (Fig. S9A). By applying the Tet-On system to replenish KANK2 expression upon doxycycline induction, we found that restoration of KANK2 partially reversed the adverse effects of USP39 knockdown on HCC cell proliferation and cell cycle control (Figs. 5G, S9B, D, F), underscoring the importance of USP39-regulated KANK2 splicing in hepatocarcinogenesis. To provide further evidence, two Tet-On shRNAs specifically targeting KANK2-L and KANK2-S were introduced into USP39-overexpressing HCC cells. KANK2-L depletion markedly suppressed the pro-proliferation phenotype of USP39, whereas KANK2-S silencing had minor effects (Figs. 5G, S9C, E, F). WB analysis confirmed that KANK2-L depletion correspondingly reduced KANK2 protein expression, while KANK2-S targeting barely affected KANK2 protein expression, despite successful knockdown of KANK2-S mRNA (Fig. S10). These data suggested that USP39 exerts its malignant effects partially through an isoform switch from KANK2-S to KANK2-L, with a shift in translation of KANK2 from low efficiency to high efficiency.

### USP39 selectively regulates exon inclusion/exclusion via interaction with SRSF6/HNRNPC in a position-dependent manner

**Fig. 6:**
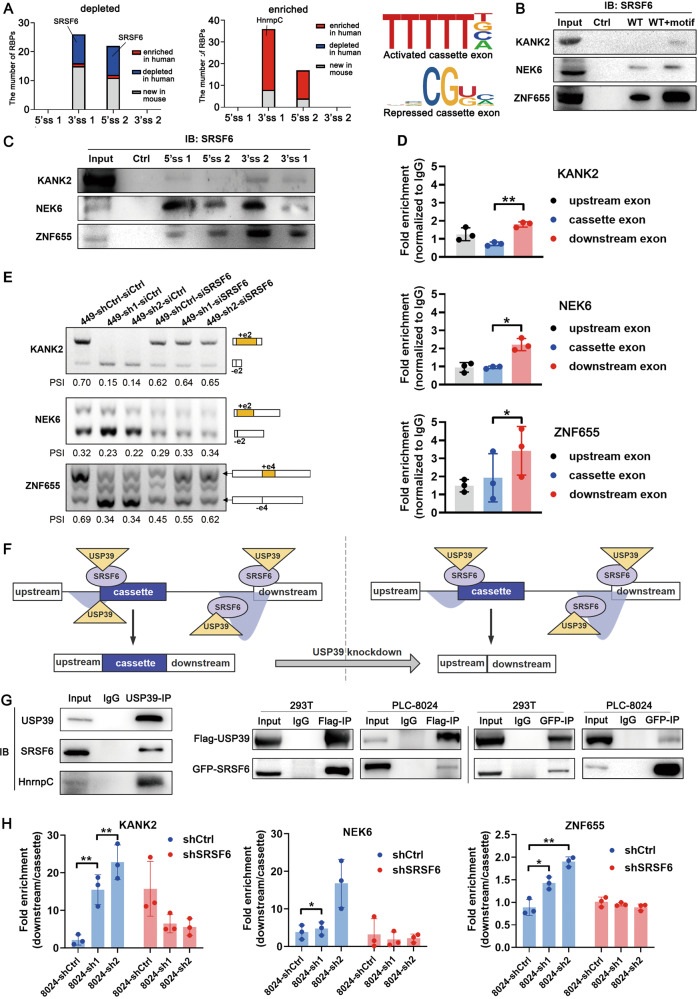
USP39 selectively regulates exon inclusion/exclusion via interaction with SRSF6 in a position-dependent manner. **A** Comparison of motif enrichment analysis results between human HCC cells and mice. The depleted SRSF6-binding motif and the enriched HNRNPC-binding motif (shown on the right panel) were distributed in a conserved pattern in humans and mice. **B**, **C** RNA pulldown assay verified the binding of SRSF6 to the putative binding motif (**B**) and predominant binding of SRSF6 within the flanking constitutive exons over the cassette exons (**C**). Representative images of three independent reproducible experiments are shown. **D** RIP assay was performed in *PLC-8024* cells with SRSF6 antibody or IgG control (*n* = 3). **E** Minigene reporters of KANK2, ZNF655, and NEK6 were introduced into USP39-knockdown *SNU-449* cells. SRSF6 was further silenced and splicing of the minigenes was verified by RT-PCR. The percentages of cassette exon inclusion within the total transcripts were presented using PSI. **F** Schematic illustration of the mechanism by which shUSP39 represses exon inclusion through interacting with SRSF6 in a position-dependent manner. **G** Endogenous (left)/ ectopic overexpressed (right) USP39 was coimmunoprecipitated with endogenous (left)/ ectopic overexpressed (right) SRSF6 protein. **H** RIP assay showed that USP39 retained in the flanking constitutive exons upon USP39 knockdown (blue columns). Silencing of SRSF6 in USP39-knockdown cells abolished USP39 retainment in the flanking constitutive exons (red columns). Data were presented as fold enrichment between downstream exon signal and cassette exon signal (downstream/cassette). Mean ± SD. *P* values were determined using paired (**D**) or unpaired (**H**) Student’s *t*-test. *P < 0.05, **p < 0.01.

Data from the mouse model suggested that USP39 modulated AS by forming a regulatory network with RBPs. We continued to perform RBP motif enrichment analysis on shUSP39-regulated exon skip events in human *PLC-8024* HCC cells. RBP-binding motifs maintained a strictly similar distribution pattern in humans and mice, including the depleted SRSF6-binding motif and the enriched HNRNPC-binding motif within the cassette exon junctions (Fig. 6A, Table S3).

The putative SRSF6-binding motif was among the top predicted motifs derived from the shUSP39-repressed exon set. The above-mentioned three shUSP39-repressed AS events (KANK2, NEK6, and ZNF655) all contained SRSF6-binding motifs in the flanking constitutive exon junction, but not in the cassette exon junction. Therefore, we investigated whether SRSF6 participates in shUSP39-repressed exon splicing. To this end, an RNA pulldown assay was performed to examine whether SRSF6 binds to this putative binding site. Biotin-labeled cassette exon RNAs (WT) were transcribed in vitro and the putative SRSF6-binding motif was inserted into these RNAs (WT+motif). Compared with WT RNAs, WT+motif RNAs pulled down more SRSF6 protein in all three tested genes (Fig. 6B), indicating that SRSF6 can recognize and bind to this putative SRSF6-binding motif. Thereafter, these splice sites in cassette exons together with the upstream and downstream constitutive exons were compared for their SRSF6 binding affinities using an RNA pulldown assay. The SRSF6 binding pattern was consistent with the motif distribution feature of shUSP39-repressed exons, which was characterized by the predominant binding of SRSF6 within the flanking constitutive exons over the cassette exons (Fig. 6C). Consistently, the RIP assay showed enrichment of SRSF6-bound flanking constitutive exon RNAs over cassette exon RNAs (Fig. 6D). To gain mechanistic insights into shUSP39-repressed exon inclusion, we constructed minigene reporters of KANK2 exons 1–3, NEK6 exons 1–3 and ZNF655 exons 3–5, respectively. Splicing was assayed following transient transfection of the control and USP39-knockdown sub-cell lines. In accordance with the endogenous splicing pattern (Fig. 4F), the inclusion of KANK2 exon 2, NEK6 exon 2, and ZNF655 exon 4 was markedly inhibited by USP39 knockdown (Fig. 6E, lanes 1–3). On this basis, siRNA-mediated SRSF6 silencing substantially restored the inclusion of cassette exons (PSI values) in the USP39-knockdown sub-cell line (Fig. 6E, lanes 5–6) to a level equivalent to that in the control sub-cell line (Fig. 6E, lane 4), indicating that the shUSP39-repressed inclusion of KANK2, NEK6, and ZNF655 cassette exons was SRSF6 dependent. Based on the above results, we proposed a model in which the enrichment of SRSF6-binding motifs within the flanking constitutive exons recruits SRSF6 and subsequently detains relatively more USP39 during USP39 knockdown, resulting in repressed cassette exon inclusion (Fig. 6F). Supporting this, SRSF6 protein was coimmunoprecipitated with USP39 in human embryonic kidney *293T* cells and *PLC-8024* HCC cells, suggesting that SRSF6 can interact and help recruit USP39 (Fig. 6G). This binding between SRSF6 and USP39 aids the recruitment of USP39 to the SRSF6-binding motif-enriched region. As a result, RIP analysis showed that USP39 was retained in the flanking constitutive exons upon USP39 knockdown, whereas SRSF6 silencing abolished USP39 retention (Fig. 6H).

**Fig. 7:**
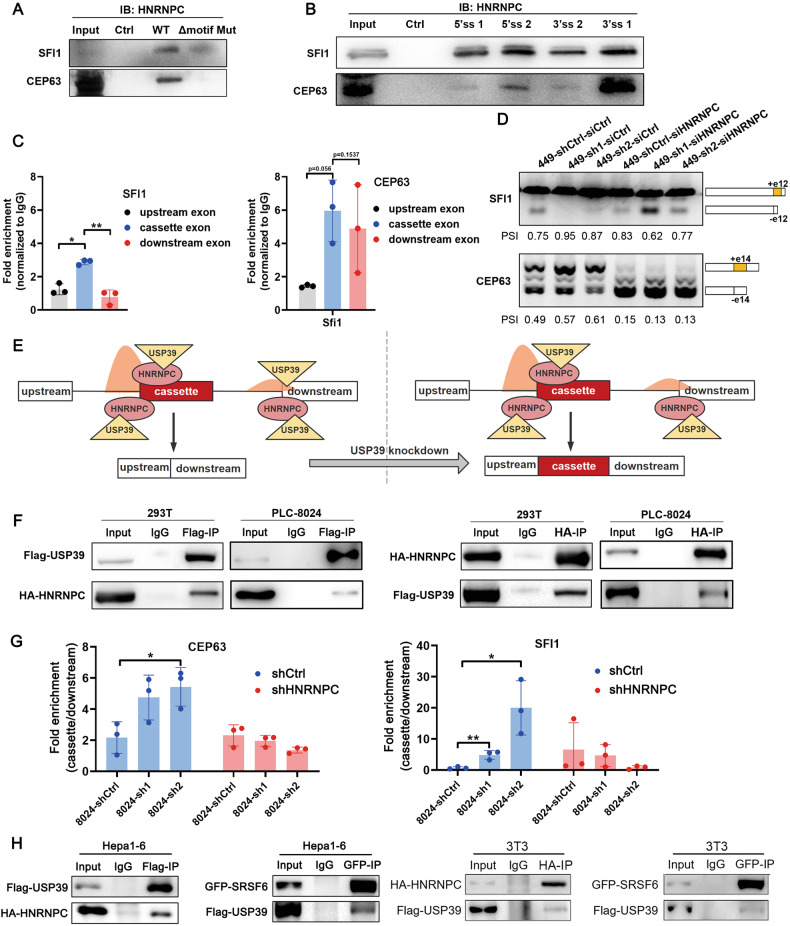
USP39 selectively regulates exon inclusion/exclusion via interaction with HNRNPC in a position-dependent manner. **A**, **B** RNA pulldown assay verified the binding of HNRNPC to the putative binding motif (**A**) and the predominant binding of HNRNPC within the cassette exons over the flanking constitutive exons (**B**). Representative images of three independent reproducible experiments are shown. **C** RIP assay was performed in *PLC-8024* cells using antibodies against HNRNPC or IgG control. The levels of precipitated cassette exon RNA and the flanking exon RNA were detected using qRT-PCR and the data were presented as fold enrichment relative to IgG control (*n* = 3). **D** Minigene reporters of SFI1 and CEP63 were introduced into USP39-knockdown *SNU-449* cells. HNRNPC was further silenced and splicing of the reporters was verified by RT-PCR. The percentages of cassette exon inclusion within the total transcripts were presented using PSI. **E** Schematic illustration of the mechanism by which shUSP39 activates exon inclusion through interacting with HNRNPC in a position-dependent manner. **F** 293T and PLC-8024 cells were co-transfected with a combination of two plasmids expressing Flag-USP39 and HA-HNRNPC proteins. co-IP experiments with anti-Flag or anti-HA agarose antibodies and visualized by WB analysis using anti-Flag and anti-HA antibodies, respectively. **G** RIP assay using USP39 antibody showed that USP39 retained in the cassette exons upon USP39 knockdown (blue columns). Silencing of HNRNPC in USP39-knockdown cells abolished USP39 retainment in the cassette exons (red columns). Data were presented as fold enrichment between the cassette exon signal and downstream exon signal (cassette/downstream). **H** Ectopic overexpressed Flag-USP39 was coimmunoprecipitated with ectopic overexpressed HA-HNRNPC/GFP-SRSF6 proteins in *Hepa1-6* and *3T3* cells. Mean ± SD. *P* values by paired (**C**) or unpaired (**G**) Student’s *t*-test. *P < 0.05, **P < 0.01.

Similarly, we investigated the mechanism by which shUSP39 activates cassette exon inclusion. The motifs derived from the shUSP39-activated exons indicated a predominant enrichment of HNRNPC binding motifs within the cassette exons over the flanking constitutive exons. Putative HNRNPC-binding motif and two shUSP39-activated AS events (SFI1 and CEP63) were selected to demonstrate the underlying mechanism. The RNA pulldown assay confirmed binding between HNRNPC and the putative binding motif (WT), which was almost abolished when the motif was deleted (Δmotif Mut) (Fig. 7A). Both the RNA-bound HNRNPC and HNRNPC-bound RNA patterns were consistent with the motif distribution features of the shUSP39-activated exons (Fig. 7B, C). Minigene reporter assays showed that the shUSP39-activated inclusion of SFI1 and CEP63 cassette exons was HNRNPC-dependent (Fig. 7D). Based on the above results, we propose a model in which the enrichment of HNRNPC-binding motifs within the cassette exons recruits HNRNPC and subsequently detains relatively more USP39 during USP39 knockdown, which results in activated cassette exon inclusion (Fig. 7E). Supporting this, endogenous/ectopically overexpressed USP39 was coimmunoprecipitated with endogenous/ectopic overexpressed HNRNPC protein, suggesting that HNRNPC can interact and help recruit USP39 (Figs. 6G, 7F). RIP analysis confirmed that USP39 was retained in HNRNPC-binding motif-enriched cassette exons upon USP39 knockdown, which was abrogated after HNRNPC silencing (Fig. 7G). Moreover, the Co-IP assay also detected interaction between USP39 and SRSF6/HNRNPC in various mouse cell lines, suggesting this regulatory network is conserved between humans and mice (Fig. 7H). An additional GST-pulldown assay was conducted to investigate the interaction manner, and the findings indicated a direct interaction between HNRNPC and USP39, while SRSF6 interacted indirectly with USP39 (Fig. S11). Collectively, these results demonstrated that USP39 selectively regulates exon inclusion/exclusion via interaction with SRSF6 or HNRNPC in a position-dependent manner.

## Discussion

Spliceosome is one of the most complex molecular machinery of the cell. At present, models of splicing are mainly delineated by detailed biochemical studies using yeast or a small number of model introns [30]. Considering the close relationship between AS and diseases including cancer, in vivo models and data are especially needed to refine the existing regulatory networks. In the current study, we profiled the global AS landscape of HCC in wild-type and USP39 conditional overexpression transgenic mice. Overexpression of USP39 significantly causes AS changes of higher magnitude in mice. This great effect on splice site selection implies that the standard models of AS regulation may underweight contributions from mid- or late-acting spliceosome proteins. Data from human HCC cells in vitro also support this notion by providing more detailed biochemical evidence. Although USP39 has been demonstrated to regulate cell activities via different mechanisms, including changing constitutive splicing efficiency and deubiquitinating protein substrates [24, 25, 31–33]. We found that USP39 promoted hepatocarcinogenesis in a spliceosome-dependent manner. Supporting this, only WT USP39, but not C139A USP39 (a mutant that impairs spliceosome assembly), rescued the malignant phenotypes of USP39-silenced HCC cell lines. Moreover, the ubiquitination of several DAS target genes regulated by USP39 did not exhibit significant alterations (Fig. S12). DAS events affected by USP39 participate in a wide range of tumor-related functions such as cell cycle control. Splicing switch of *KANK2* has been demonstrated to be an important molecule that mediates the oncogenic effects of USP39 in HCC. These findings suggest that mid/late-acting spliceosome component USP39 is a potent regulator of AS and the resulting isoform switching is functionally important. Of note, our data do not rule out other possible mechanisms. In fact, USP39 knockdown also reduces global constitutive splicing efficiency, which is tightly coupled with transcription and gene expression.

Previous studies have mainly stated the role of U1 and U2 snRNP components in splice site selection. However, how can late-acting spliceosome components such as USP39 influence splice site choices? Substantial studies have provided a consensus picture in which splicing factors recognizing cognate auxiliary sequences in the pre-mRNA promote or inhibit early events in spliceosome assembly (e.g., U1 snRNP and U2 snRNP recruitment) [34]. These regulatory factors include members of hnRNP and SR protein families, which often display cooperative or antagonistic functions depending on the position of their binding sites relative to the regulated splice sites [35, 36]. In our study, we proved that these hnRNP and SR proteins also regulated the recruitment of U4/U6. U5 tri-snRNP components, thereby influencing specific splice site recognition. Based on the mechanistic investigations, including motif analyses, RNA pulldown assays, RIP, and minigene reporter assays, we found that depletion of SRSF6-binding motifs in the cassette exons always led to exon exclusion, whereas enrichment of HNRNPC-binding motifs within the cassette exons always led to exon inclusion upon USP39 knockdown. Intriguingly, this mechanism is conserved in mice and humans. Although a considerable fraction of AS events appear to be species-specific, the interaction between USP39 and SRSF6/HNRNPC is conserved between humans and mice. It is conceivable that such RBPs would favor the splicing of alternative splice sites they recognize by recruiting limited amounts of the spliceosome component USP39. In this context, regulatory plasticity may be attributed, at least in part, to the substantial number of splicing factors containing disordered regions. USP39, SRSF6, and HNRNPC contain intrinsically disordered regions [37–39]. Such regions may be flexible to adopt different conformations, allowing alternative routes for spliceosome assembly on different introns, with some splice sites being more sensitive than others to the depletion of a core spliceosomal factor [40].

Besides SRSF6 and HNRNPC, RBP-binding motif enrichment analyses also revealed that other RBPs are potentially involved in USP39-modulated splicing. And there are nearly 100 mid- or late-acting spliceosome components in addition to USP39, which reminds us about the extraordinary complexity of AS regulatory networks. The RBP-binding motif enrichment analyses we developed in this study illustrate the potential to identify molecular mechanisms of regulation on the basis of RNA-seq data. There is now a wealth of publicly available RNA-seq datasets generated upon the knockdown of hundreds of RNA-related genes as part of the ENCODE project. This may provide abundant bio information for us to systematically construct the AS networks. Furthermore, both the direct-interacting proteins (such as HNRNPC) and indirect-interacting proteins (such as SRSF6) have impacted USP39’s AS-regulating preferences. Consequently, protein-interaction assays including GST-pulldown, are still necessary to assess the directness of the interactors and verify the AS networks.

In summary, the AS landscape profiled in mouse HCC highlights the remarkable regulatory potential of USP39 in splice site selection. USP39 is a driving factor for hepatocarcinogenesis, whose function is carried out in a spliceosome-dependent manner and at least partially mediated by the oncogenic splicing switch of *KANK2* gene. Our analysis provided a new regulatory model that is conserved in humans and mice, whereby USP39 regulates exon inclusion/exclusion by interacting with SRSF6 or HNRNPC in a position-dependent manner. These findings refine the standard model by demonstrating mid/late-acting spliceosome components can regulate splice site selection through cooperation with other splicing regulators. USP39 and possibly other mid/late-acting spliceosome components may serve as tumor biomarkers and therapeutic targets. As a general approach, our data suggest that systematic identification of AS targets can be combined with bioinformatics and biochemistry to identify the sites and mechanisms of action of alternative splicing factors.

## Materials and methods

### Clinical samples

66 pairs of HCC and para-tumor tissue samples were collected with informed consent from patients who underwent hepatectomy at Sun Yat-Sen University Cancer Center (Guangzhou, China). The clinical specimens used in this study were approved by the Committee for Ethical Review of Research Involving Human Subjects at the Sun Yat-Sen University Cancer Center (GZR2020-260). Complete clinicopathological and follow-up data were available and the patient demographic information is provided in Table S4.

### Animal experimentation

All animal experiments were reviewed and approved by the Institutional Animal Care and Use Committee, Southern University of Science and Technology (SUSTC-2019-069). All mice had a C57bl/6 background. *Usp*39^HOE^ mice were obtained by crossing *Rosa26-stopflox/flox-USP39* (Cyagen Biosciences, Guangzhou, China) and *Albumin-Cre* (Shanghai Model Organisms Center, Shanghai, China) mice. The resulting *Rosa26-stopflox/flox-USP39*; *Alb-Cre* mice served as the experimental group and *Alb-Cre/+* mice served as a control group. Only the male mice were used in this study.

To establish the HCC model, intraperitoneal injection of 20 mg/kg diethylnitrosamine (DEN) (Sigma, #N0258-1g, MO, USA) was performed within 2 weeks after birth to initialize the HCC process. CCl4 (2 μl/g body weight, prediluted 1:10 in olive oil) was intraperitoneally injected twice a week after an interval of 6 weeks to promote HCC progression. The mice were sacrificed after 16-week promotion and the liver/body weight, tumor number, and expression levels of CCND1 and Ki67 were assessed.

### RBP motif enrichment analysis

For RBP motif enrichment analysis, a reference sequence was downloaded from the ATtRACT database. The background sequences were generated by randomly selecting 150,000 exons from the GRCh38 or GRCm39 reference genome and retrieving DNA sequences of their 5’ and 3’ splice sites (i.e., regions with a length of 100 bp flanking the 5’ and 3’ ends of these exons). The numbers of RBP motifs in splice sites associated with significant alternative splicing events and the random background sequences were counted using HOMER and then used in enrichment analysis of these motifs based on one-sided Fisher’s exact-test (left-sided test for identification of deprived motifs and right-sided test for enriched motifs) implemented in MATLAB scripts. *P*-values were adjusted using the Benjamini–Hochberg procedure for multiple hypothesis testing.

### Supplementary information

Supplementary files

Table S1

Table S2

Table S3

Table S4

Table S5

aj-checklist-revised

## Data Availability

The data generated in this study are publicly available in the Sequence Read Archive (SRA) database at PRJNA855898 and PRJNA864041. The data of mass spectrometry analysis generated in this study are publicly available in the PRIDE database at PXD035928.

## Code availability

Code is available on GitHub at https://github.com/VikkiYan/RBP-enrichment.git.
