# Supplementary material for: USP39 promotes hepatocellular carcinogenesis through regulating alternative splicing in cooperation with SRSF6/HNRNPC
Source: Cell Death Dis. 2023 Oct 11;14(10):670. doi: 10.1038/s41419-023-06210-3 (PMC10567755; doi:10.1038/s41419-023-06210-3)
Supplement: Supplementary file 1 — Supplementary files [file 41419_2023_6210_MOESM1_ESM.pdf]

Supplementary Methods

Consensus clustering for proteomic data

HCC proteomic data were retrieved from the Clinical Proteomic Tumor Analysis Consortium (CPTAC). Consensus clustering was implemented for 1,274 differentially expressed proteins (1) using the ConsensusClusterPlus R package, and the following parameters were used for clustering: number of repetitions = 1,000 bootstraps; pItem = 0.8 (resampling 80% of any sample); pFeature = 0.8 (resampling 80% of any protein); and k-means clustering with up to 6 clusters, and 3-cluster as the optimized solution for clustering. Gene Ontology (GO) and Kyoto Encyclopedia of Genes and Genomes (KEGG) enrichment analyses were performed using the online tool MSigDB (https://www.gsea-msigdb.org/gsea/msigdb/).

Survival-related analyses

Using the GEPIA2 webserver (http://gepia2.cancer-pku.cn/), the association between each spliceosomal gene (2) expression level and overall survival (OS) for all cancers was determined by univariate Cox regression for the highly expressed group versus the low one, with the median expression as a cut-off value. The log-rank test was used to compare differences in survival distribution.

TCGA and GEO data analyses

The HCC transcriptome data were obtained from The Cancer Genome Atlas

Liver Hepatocellular Carcinoma (TCGA_LIHC) project and the NCBI Gene Expression Omnibus (GEO) database (accession no. GSE124535, GSE14520). Gene Set Enrichment Analysis (GSEA) (http://www.broadinstitute.org/gsea) was performed to identify associated molecular pathways.

Western Blot

Samples were lysed with Pierce RIPA buffer (Thermo Scientific, Massachusetts, USA) and quantified using Bio-Rad Protein Assay (Bio-Rad, California, USA). After being mixed with loading dye (Bio-Rad), protein was denatured (100℃, 15min). Denatured proteins were separated by SDS-PAGE and transferred onto a PVDF membrane (Millipore, Massachusetts, USA). After blocking, the membranes were incubated with primary antibodies (USP39, Abcam, Cambridge, UK, #ab131244; CCND1, CST, Massachusetts, USA, #2978; CDK2, CST, #2546; CDK6, CST, #3136; β-actin, CST, #3700; GAPDH, CST, #2118; KANK2, Proteintech, Illinois, USA, # 21733-1-AP; SRSF6, ABclonal, Massachusetts, USA, #A14603; HNRNPC, Proteintech, #11760-1-AP; Flag, Proteintech, #20543-1-AP; GFP, RUIXIN, Quanzhou, China, # GXP204228; HA, CST, #3724) and incubated with peroxidase-conjugated secondary antibodies. Immunoreactive bands were visualized using ECL (Bio-Rad) and exposed to autoradiograph film.

Immunohistochemistry

Liver tissues were fixed in 4% paraformaldehyde, embedded in paraffin, and sectioned at 5 μm thickness for IHC staining. After blocking with 5% bovine serum albumin, the deparaffinized sections were incubated with primary antibodies (Ki67, CST, #12202; CCND1, CST, #2978) overnight at 4 ˚C. Antibody binding was detected by incubation with biotinylated anti-rabbit IgG antibody and visualized by reaction with DAB Substrate (Boster, California, USA).

Cell lines

The HCC cell line *PLC-8024* (TCHu119) was obtained from the Institute of Virology of the Chinese Academy of Sciences (Beijing, China). The HCC cell line *SNU-449* (CRL-2234) was obtained from the American Type Culture Collection (ATCC). Cell line *293T* (SCSP-502) was obtained from the Cell Bank affiliated to Shanghai Institute of Biochemistry and Cell Biology. The mouse HCC cell line *Hepa1-6* (CTCC-ZHYC-0566) was obtained from Meisen Chinese Tissue Culture Collections (MeisenCTCC). The mouse embryo fibroblast cell line *3T3* was a kind gift from Dr. Xijun Ou. All cell lines were authenticated using short tandem repeat profiling and routinely tested for mycoplasma contamination. The cells were cultured in Dulbecco’s modified Eagle’s medium (DMEM) (Gibco, California, USA), supplemented with 10% fetal bovine serum (Gibco), and 1% penicillin/streptomycin mixture (Gibco). All cell lines used in this study were incubated at 37°C in a humidified incubator containing 5% CO2.

siRNA transfection

siRNA sequences were designed using the siDRECT website (http://sidirect2.rnai.jp/) and the sequences are listed in Table S5. siRNA transfection was performed using siRNA-mate reagent (GenePharma, Shanghai, China) according to the manufactures' instruction.

Plasmids, lentivirus production and cell infection

Wild-type and mutant (C139A) USP39 with a Flag-tag at its N-terminal were cloned into the pLenti6/V5 lentiviral vector (Invitrogen, California, USA). Specific shRNA oligonucleotides targeting USP39 were cloned into the pLL3.7 lentiviral vector (Addgene). These plasmids, together with lentivirus packaging vectors from the pLenti6/V5 Directional TOPO Expression Kit (Invitrogen), were co-transfected into *HEK293T* cells using Lipo3000 (Invitrogen). After 3-day incubation, the medium containing the specific virus was collected and added to *PLC-8024* and *SNU-449* cells combined with 10μg/ml polybrene. After 72 h, pLenti6/V5-Flag-USP39 infected cells were selected with 5 μg/ml blasticidin (Gibco) while pLL3.7-shRNA infected cells were selected with 2μg/ml puromycin (Gibco). The overexpression and knockdown efficiencies were validated with WB and qRT-PCR. primers and USP39-shRNAs sequences are listed in Table S5.

Tet-On system mediated overexpression and knockdown

Tet-On 3G systems are inducible gene expression systems with two elements,

namely, the Tet-On 3G transactivator protein and a gene of interest under the control of a TRE3G promoter. The PiggyBac transposon system has been shown to be highly efficient in mediating gene transfer.(3) Therefore, it has been modified to deliver the multiplex Tet-On 3G system. Briefly, the KANK2 gene sequence and two shRNAs targeting the KANK2-L isoform (shKANK2-L) and KANK2-S isoform (shKANK2-S) were cloned into the pBX-093 plasmid (PB5-HS4-SV40-puro-2A-tetON3G-pA-HS4-TRE-AzaminGreen-2A-Tet3G-RNAi-GpA-HS4-PB3, a kind gift from Dr. Wei Huang, Southern University of Science and Technology, Shenzhen, China) respectively. Cells were subsequently co-transfected with the constructed pBX-093 plasmid and pBX-090 plasmid (pN1-CMV-PGK-piggybac, a kind gift from Dr. Wei Huang) and sorted based on Azamin Green. Before the functional assays, cells were treated with 1μg/ml doxycycline (DOX) to induce the overexpression of KANK2 or knockdown of KANK2-S and KANK2-L. Primers and KANK2-shRNAs sequences are listed in Table S5.

RNA extraction, PCR and qRT-PCT analysis

RNA extraction was performed using TRIzol reagent (Vazyme Biotech, Nanjing, China) according to the manufacturer's instructions. 1μg of total RNA was used to synthesize the first strand of cDNA using Hifair® Ⅲ 1st Strand cDNA Synthesis SuperMix (Yeasen, Shanghai, China, #11141ES60,).

For PCR analysis, Green Taq Mix (#P131-03, Vazyme) was used according to the manufacturer's instructions. PCR products were analyzed by

electrophoresis on a 1% agarose gel.

For qRT-PCR analysis, the SYBR® Green Premix Pro Taq HS qPCR Kit (Accurate Biology, Changsha, China, #AG11701) was used according to the manufacturer's instructions. The relative changes in gene expression were calculated using the 2 -ΔΔCt method. The primer sequences are listed in Table S5.

Cell viability assay, foci formation assay and soft agar colony formation assay

CCK8 assay was performed to analyze cell viability. Cells were digested and re-cultured in 96-well plates at 2000 cells per well in 100μl of medium. Each well was added 10μl CCK8 solution (MCE, New Jersey, USA, HY-K0301) and incubated for 3 h at 37 °C. Optical density was measured using a microplate reader at a wavelength of 452 nm within 5 days. Triplicate repeats were performed to determine the variance and significance. The values were plotted by averaging triplicate wells.

For the foci formation assay, cells were digested and re-cultured in 6-well plates at 500 cells per well in 2 ml medium. After incubation for 9 days, natural monolayer colonies were formed. The cells were subsequently washed with PBS, fixed with 4% paraformaldehyde, stained with purple crystals for 10 min, and washed with PBS three times. The results were photographed, and the number of clones was counted.

For the soft agar colony formation assay, 2000 *PLC-8024* cells were mixed

with 10% FBS, 1× DMEM, and 0.35% agar as the upper layer, whereas the bottom layer contained 10% FBS, 1× DMEM and 0.6% agarose. The colonies were photographed under a microscope and the clone numbers were counted 20 days later.

Cell cycle flow cytometry

Cells were grown to a density of 70% in 6-well dishes and collected for staining with propidium iodide (PI, Sangon, Shanghai, China, #A601112-0100) according to the manufacturer's instructions. A CytoFLEX cytometer (Beckman Coulter, California, USA) was used to measure changes in cell cycle, and Modfit software was used to analyze the results.

A double thymidine block was used to synchronize cells at the G1/S boundary. Briefly, the cells were treated with 2mM thymidine (Thd, Sigma, #T1895-1G) for 18h and then washed to remove thymidine. After a 9-hour incubation in fresh medium, the cells were treated with the second round of Thd (2 mM) and synchronized at the G1/S boundary. Cells were released by washing with pre-warmed 1x PBS and incubating the cells in pre-warmed fresh media. Cells were collected at 0, 3h for analysis of cell cycle by DNA staining using PI.

To synchronize the cells at the G2/M boundary, the cells were treated with 2mM Thd for 24h and then washed to remove thymidine. After 3-hour incubation in fresh medium, cells were successively treated with 100ng/ml nocodazole (MCE, #HY-13520) and then synchronized at the G2/M boundary. Cells were

released by washing with pre-warmed 1x PBS and incubating the cells in pre-warmed fresh media. Cells were collected at 0, 4h for analysis of cell cycle by DNA staining using PI.

Biotinylated RNA pulldown and RNA pulldown coupled with mass spectrometry

Biotinylated RNA-pulldown assays were performed as previously described(4). Briefly, exon with 60bp intron sequences at the 5'/3' splice site were cloned into pcDNA3.1, and the plasmid was linearized with the following primers: Forward: TGCTCTGATGCCGCATAGTT, Reverse: GCCCACTACGTGAACCATCA. The linearized DNA fragment was used as a template to generate biotin-labeled RNA for precipitation from *PLC-8024* cell lysate. Subsequently, WB assay was performed to detect precipitation.

RNA pulldown coupled with mass spectrometry was performed as previously described.(5) Briefly, biotinylated DNA probes were designed to target the exon1-2 junction or the exon1-3 junction of KANK2 and their antisense sequences (exon1-2 sense: CAGCGGCCGGAGCGCGCAAGGTGTTGAAAGACAGAGAAGC, exon1-3 sense: CAGCGGCCGGAGCGCGCAAGGTAAGCCTCAGCCGGTGCTG, exon1-2 antisense: GCTTCTCTGTCTTTCAACACCTTGCGCGCTCCGGCCGCTG, exon1-3 antisense: CAGCACCGGCTGAGGCTTACCTTGCGCGCTCCGGCCGCTG). These probes were incubated with *PLC-8024* cell lysates, followed by an

additional incubation with streptavidin beads. After on-bead digestion and desalination, the extracted peptides were analyzed by LC-MSMS.

RIP

A total of 107 cells were washed with PBS and crosslinked with a 1% formaldehyde solution for 15 min. After neutralization with 0.125M glycine, the cells were lysed in RIPA lysis buffer (Week) (Beyotime Biotechnology, Shanghai, China, #P0013D) for 15 min and ultrasonicated. 0.5μg mouse anti-Flag antibody (Sigma, #F1804) or IgG control (Mouse IgG, Sigma, #I5381-1MG; Rabbit IgG, ThermoFisher, # 10500C) was used for immunoprecipitation. A 10%-volume of lysate was used as the input. RNA in the precipitates and input was subsequently extracted using the acid phenol-chloroform method. The extracted RNAs were detected by qRT-PCR using the Universal Blue qPCR SYBR Green Master Mix (Yeasen, #11184ES08) according to the manufacturer’s instructions. The expression of RIP RNA was calculated as follow(6): %input(RIP)=$2^{-\Delta Ct(\text{normalized RIP})}$. ΔCt(normalized RIP)=Average Ct(RIP) - Average Ct(input) - log2(input dilution factor), input dilution factor=(fraction of input RNA)$^{-1}$. The data were presented as fold enrichment of RNAs in RIP over the IgG control: fold enrichment=% input(RIP)/ % input(IgG)

RNA seq samples preparation and analysis

USP39 knockdown and control *PLC-8024* cells (three biological replicates per sample) and the tumor or para-tumor tissues collected from *Usp*39^{HOE} and wild-

type (WT) mice were suspended in TRIzol reagent (Invitrogen) and sent to Novogene for RNA sequencing. RNA-seq libraries prepared using oligo (dT) beads and rRNA removal methods were pooled and sequenced using an Illumina platform. Paired-end reads were mapped to the Homo sapiens GRCh38(hg38) transcriptome and the Mus musculus reference genome GRCm39(mm39) using the STAR RNA-seq aligner. Alignment files were filtered and sorted using SAMTools. The number of reads mapped to each gene was quantified based on the processed alignment files using htseq-count, and differential expression analysis was performed using DESeq2. Genes with a Padj value < 0.05 and |log2(fold change)|>1 were determined to be differentially expressed. GO and KEGG enrichment analyses were performed using the online tool MSigDB (https://www.gsea-msigdb.org/gsea/msigdb/).

Analysis of pre-RNA splicing efficiency was performed based on the processed alignment files. Briefly, paired-end reads were aligned to the human reference genome GRCh38 using the STAR aligner, and then filtered and sorted using SAMTools. For each splice site, the number of reads covering the first base at the 5’ end of the intron (i.e., 5’ intron coverage), number of reads covering the last base at the 3’ end of the intron (i.e., 3’ intron converage), number of reads covering the last base at the 3’ end of the upstream exon (i.e., 3’ exon coverage), and number of reads covering the first base at the 5’ end of the downstream exon (i.e., 5’ exon coverage) were quantified using bedtools. Splicing efficiencies at the 5’ and 3’ splice sites were then computed as follows:

Efficiency 5' = 1-(5' intron coverage)/(3' exon coverage) and, Efficiency 3' = 1 – (3' intron coverage)/(5' exon coverage). Splice sites with 5' and 3' exon coverage lower than 100 were removed from the downstream analysis.

Analysis of alternative splicing and pre-RNA splicing efficiency was performed using rMATS with default parameters. Total counts of reads spanning junctions and reads that did not cross an exon boundary (i.e., outputs in the files [AS_Event]). MATS.JCEC.txt) was used together with the counts of intron reads to identify alternative splicing events. Alternative splicing events with FDR<0.05 and |IncLevelDifference|>0.1 were identified as significant events. Events shared between different experiments were identified based on the genomic coordinates of their associated exons using MATLAB scripts. Genomic coordinates of exons involved in alternative splicing events were retrieved from the rMATS output files using awk.

Minigene and PCR assay

Upstream, cassette and downstream exons with 60 bp intron sequences at the 5' and 3' splice sites were cloned into the pcDNA3.1(-) vector plasmid. Splicing of minigenes was verified by RT-PCR. The primer sequences are listed in Table S5.

Glutathione S-transferase (GST) pull-down assay

GST-USP39 fusion protein was expressed in 293F cells and purified with Glutathione Sepharose® 4B (Cytiva, #17075601). 6×His-SRSF6/HNRNPC

fusion protein were expressed in E. coli Rosetta cells and purified with Ni-NTA agarose (Qiagen, # 30210). In GST pull-down assay, 120μg GST-USP39 protein and 30uL Glutathione Sepharose® 4B were mixed and incubated in PBS at 4℃ overnight. Subsequently, 120μg 6×His-SRSF6/HNRNPC fusion protein were mixed and incubated in PBS at 4℃ for 6h. The beads were then washed with washing buffer for 3 times and GST-USP39-HNRNPC/SRSF6 complex was eluted by elution buffer (50mM Tris-HCl, 10mM reduced glutathione). The elute mixed with 5× SDS-PAGE loading buffer was then evaluated with Western Blot assay using anti-GST (Abbkine, # ABT2030) and anti-His (TransGen, # HT501-01) antibodies.

Statistical analysis

Statistical analyses were performed using SPSS 24.0 or GraphPad Prism 8.0. The mRNA levels of USP39 and KANK2-L isoforms in paired non-tumor and tumor samples were compared using a paired two-tailed Student's t-test. *USP39* expression levels in unpaired clinical samples were compared using unpaired two-tailed Student's t-test. *USP39* expression levels in HCC samples with different tumor stages and neoplasm histologic grades were compared using the Kruskal-Wallis test. Differences in overall survival and disease-free survival were calculated using Kaplan-Meier plots and log-rank tests. Correlations between two statistical variables were analyzed using Pearson's correlation analysis. An unpaired two-tailed Student's t-test was used to compare observations, such as the number of foci and the relative expression

of target genes, between any two preselected groups. Results are expressed as the mean ± standard error of the mean. P<.05 was considered to be statistically significant.

References

1. Gao Q, Zhu H, Dong L, Shi W, Chen R, Song Z, et al. Integrated Proteogenomic Characterization of HBV-Related Hepatocellular Carcinoma. Cell. 2019;179(2):561-77.e22.
2. Seiler M, Peng S, Agrawal AA, Palacino J, Teng T, Zhu P, et al. Somatic Mutational Landscape of Splicing Factor Genes and Their Functional Consequences across 33 Cancer Types. Cell reports. 2018;23(1):282-96.e4.
3. Lu X, Huang W. PiggyBac mediated multiplex gene transfer in mouse embryonic stem cell. PloS one. 2014;9(12):e115072.
4. Panda AC, Martindale JL, Gorospe M. Affinity Pulldown of Biotinylated RNA for Detection of Protein-RNA Complexes. Bio-protocol. 2016;6(24):e2062.
5. Savulescu AF, Stoychev S, Mamputha S, Mhlanga MM. Biochemical Pulldown of mRNAs and Long Noncoding RNAs from Cellular Lysates Coupled with Mass Spectrometry to Identify Protein Binding Partners. Bio-protocol. 2020;10(11):e3639.
6. Gagliardi M, Matarazzo MR. RIP: RNA Immunoprecipitation. Methods in molecular biology (Clifton, NJ). 2016;1480:73-86.

Supplementary Figures

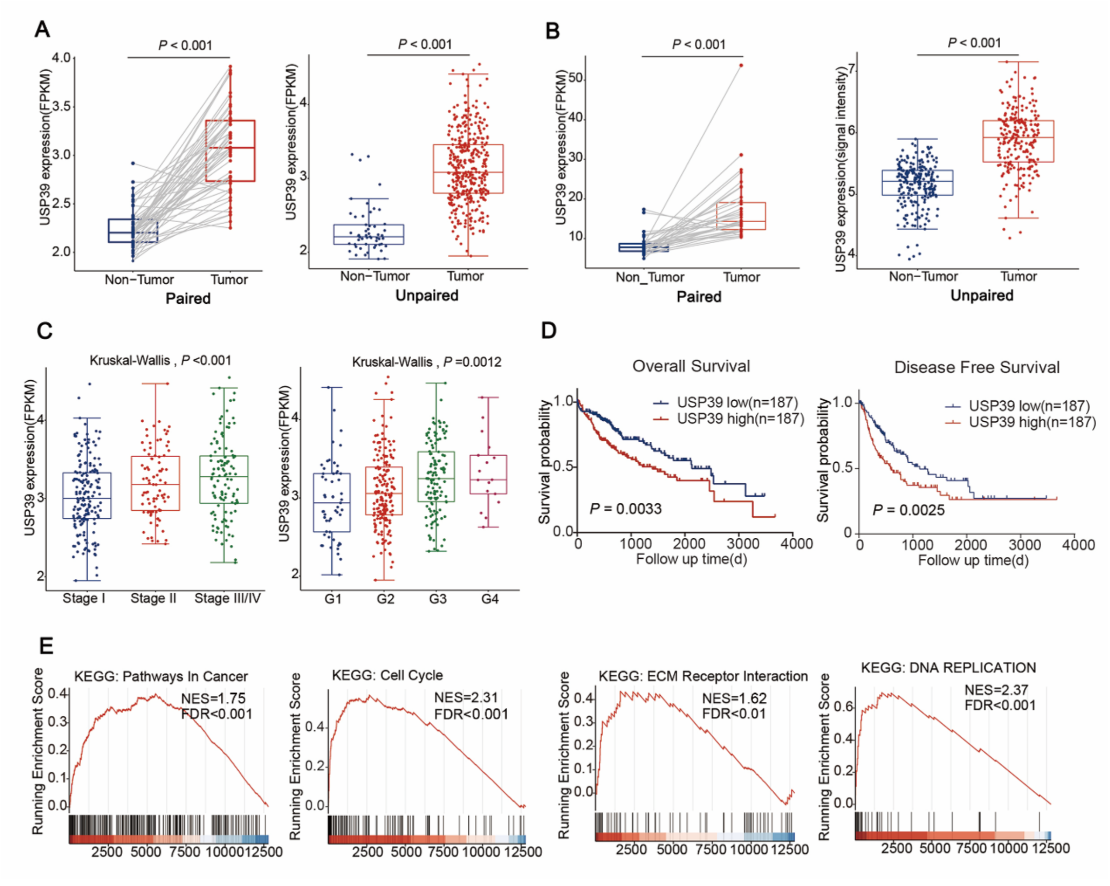

Fig. S1. Overexpression of USP39 associates with HCC pathogenesis and aberrant cell cycle signaling.

(A) USP39 expression in TCGA-LIHC database, paired and unpaired Student's t-test.

(B) USP39 expression was upregulated in paired (accession no. GSE124535) and unpaired HCC tissues (accession no. GSE14520) in GEO cohorts, paired and unpaired Student's t test.

(C) mRNA levels of USP39 in HCC samples with different tumor stages and neoplasm histologic grades (TCGA-LIHC), Kruskal-Wallis test.

(D) Kaplan–Meier OS curves and DFS curves of TCGA HCC patients with high or low USP39 expression (median expression value as a cut-off).

(E) GSEA of the indicated gene sets in USP39 high versus low patients from the TCGA-LIHC dataset.

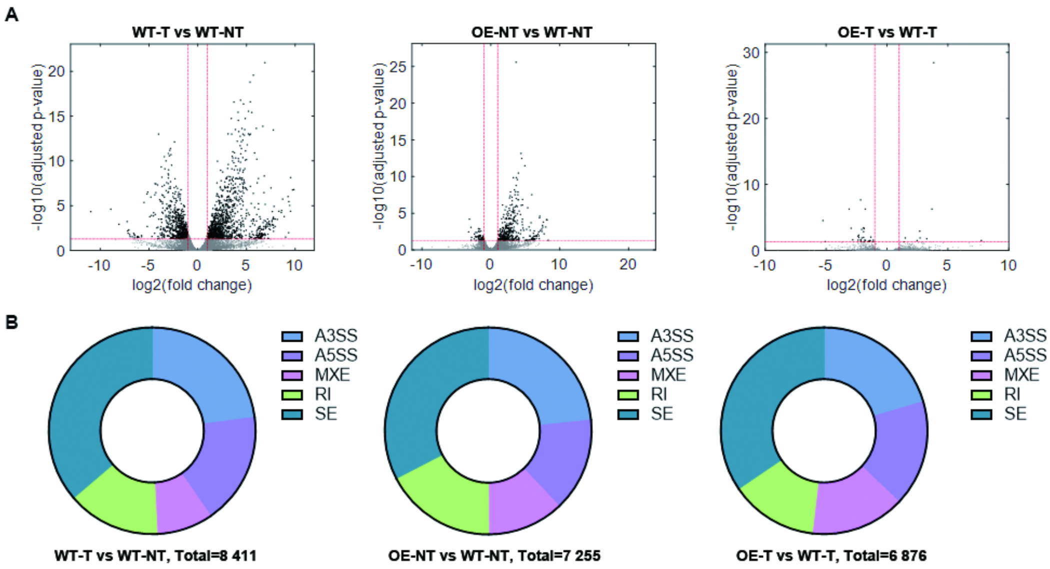

Fig. S2. Transcriptome sequencing performed using tumor (T) and para-tumor (NT) tissues of USP39HOE and WT mice.

(A) Volcano plot of DEGs in the WT-T vs. WT-NT (left), OE-NT vs. WT-NT (middle) and OE-T vs WT-T datasets (right).

(B) Pie chart depicting the proportions of DAS event types in the WT-T vs. WT-NT (left), OE-NT vs. WT-NT (middle) and OE-T vs WT-T datasets (right).

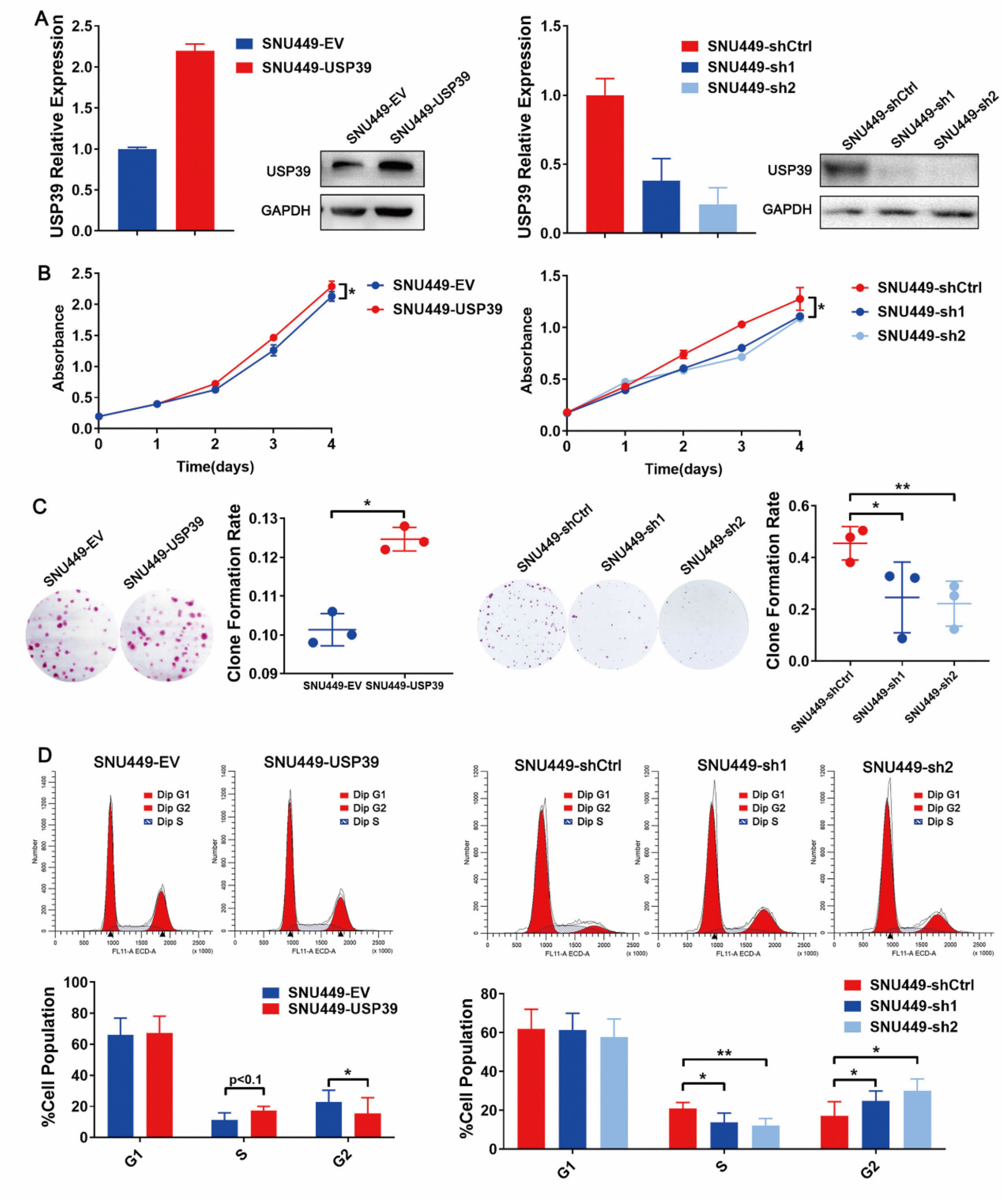

Fig. S3. USP39 promotes HCC cell proliferation and cell cycle progression in *SNU-449* cells.

(A) Ectopic expression/knockdown of USP39 in *SNU-449* cells was verified by qRT-PCR and WB (EV: Empty Vector, Ctrl: Control).

(B) CCK8 assay revealed that overexpression of USP39 significantly increased cell proliferation, while knockdown of USP39 inhibited cell proliferation.

(C) Representative images and quantification of foci formation induced by the indicated cells (n=3).

(D) Representative flow cytometry histograms of cell cycle progression and statistical results of cell cycle phase distribution (n=3).

Mean ± SD. P values by unpaired Student’s t test. ∗P <.05, ∗∗P <.01.

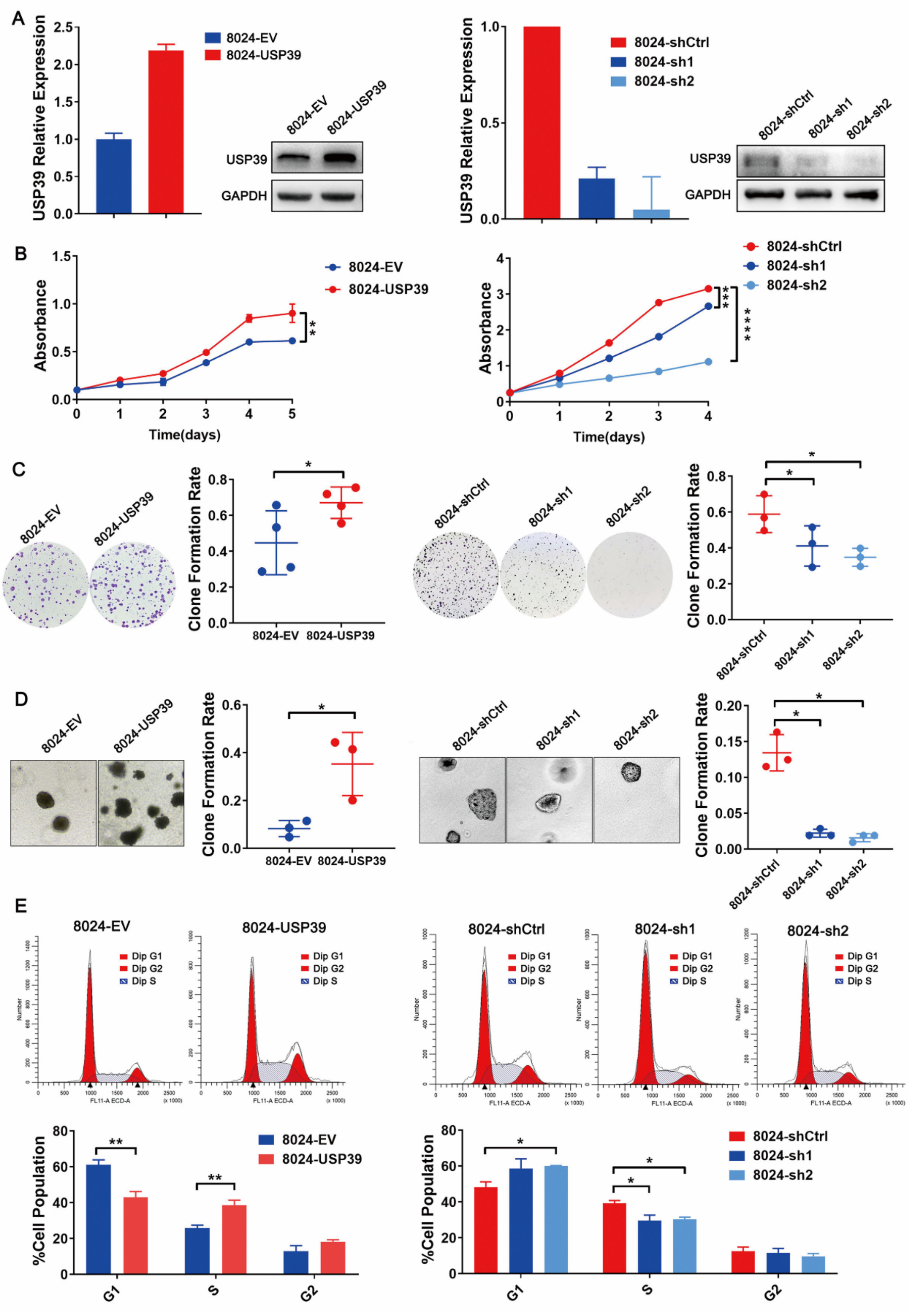

Fig. S4. USP39 promotes HCC cell proliferation and cell cycle progression in *PLC-8024* cells.

(A) Ectopic expression/knockdown of USP39 in *PLC-8024* cells was verified by qRT-PCR and WB (EV: Empty Vector, Ctrl: Control).

(B) CCK8 assay assessed viability of the indicated cells.

(C, D) Representative images and quantification of foci formation (C) or clone formation in soft agar (D) induced by the indicated cells (n=3).

(E) Representative flow cytometry histograms of cell cycle progression and statistical results of cell cycle phase distribution (n=3).

Mean ± SD. P values were determined using unpaired Student’s t-test. *P <.05, **P <.01, ***P < .001, ****P < .0001.

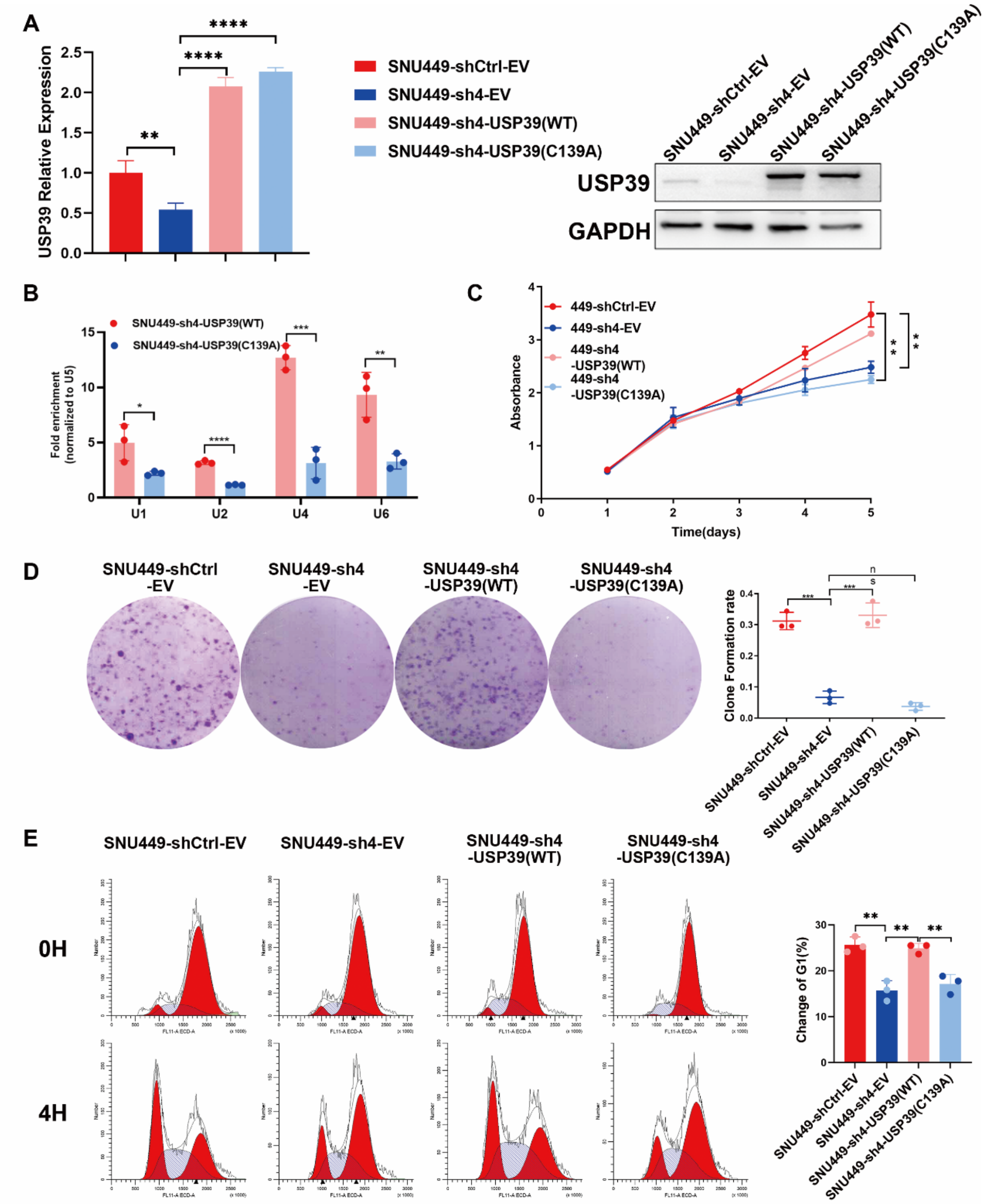

Fig. S5. Wild-type USP39, but not C139A mutant, counteracted the inhibitory effect of shRNA (sh4, which targeted the UTR of USP39) on cell cycle and proliferation in *SNU-449* cells.

(A) Flag-USP39 (WT) and Flag-USP39 (C139A) were introduced to USP39-deficient (sh4) *SNU-449* cells and validated using qRT-PCR and WB assays.

(B) RIP experiments were conducted using antibodies against Flag in the indicated cells. Levels of U1, U2, U4, U6 snRNA in immunoprecipitated RNA were detected using qRT-PCR and normalized to the levels of U5 snRNA in each sample (n=3).

(C) The CCK8 assay demonstrated that only wild-type USP39, not the C139A mutant, could counteract the inhibitory effect of USP39 deficiency on cell proliferation.

(D). Representative images and quantification of foci formation induced by the indicated cells (n=3).

(E) Cells were synchronized at the G1/S boundary and assessed with PI-stained flow cytometry after release. Cell cycle profiles showed the G2 cell population at 3 hours post-release (n=3).

Mean ± SD. P values by paired (B) or unpaired Student's t test (A, C-E). *P <.05, **P <.01, ***P < .001, ****P < .0001. ns: not significant.

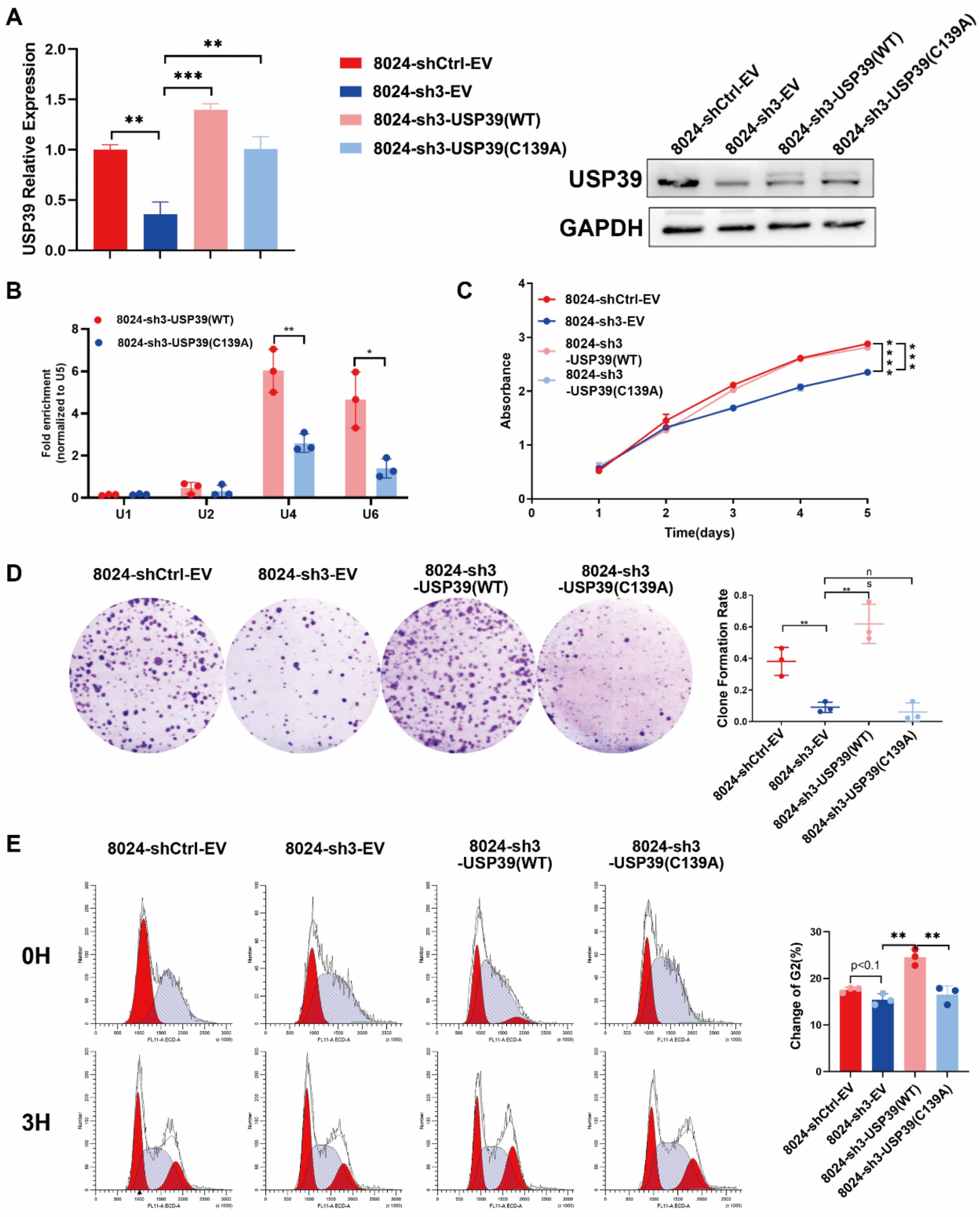
A
USP39 Relative Expression
8024-shCtrl-EV
8024-sh3-EV
8024-sh3-USP39(WT)
8024-sh3-USP39(C139A)
USP39
GAPDH
B
Fold enrichment (normalized to U5)
U1
U2
U4
U6
C
Absorbance
Time(days)
8024-sh3 -USP39(WT)
8024-sh3 -USP39(C139A)
D
Clone Formation Rate
ns
E
0H
3H
Change of G2(%)
p<0.1

346

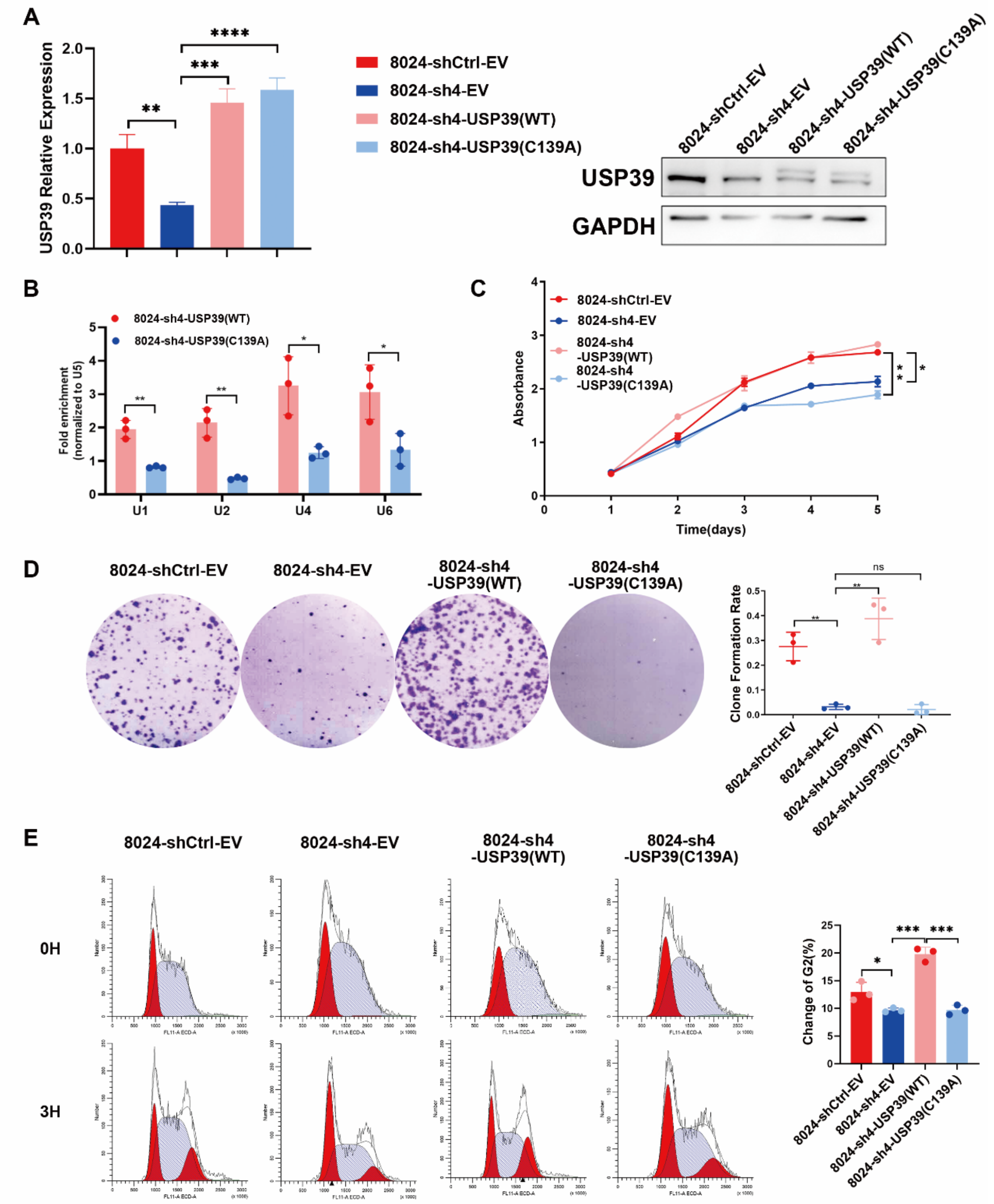

Fig. S6, 7. Wild-type USP39, but not the C139A mutant, counteracted the inhibitory effect of two shRNAs targeting the UTR of USP39 (sh3 in Figure S6 and sh4 in Figure S7) on cell proliferation in *PLC-8024* cells.

(S6A, S7A) Flag-USP39 (WT) and Flag-USP39 (C139A) were introduced to USP39-deficient (sh3 or sh4) *PLC-8024* cells and validated using qRT-PCR and WB assays.

(S6B, S7B) RIP experiments were performed using antibodies against Flag in the indicated cells. Levels of U1, U2, U4, U6 snRNA in immunoprecipitated RNA were detected using qRT-PCR and normalized to the levels of U5 snRNA in each sample (n=3).

(S6C, S7C) The CCK8 assay showed that only wild-type USP39, not the C139A mutant, could counteract the inhibitory effect of USP39 deficiency on cell proliferation.

(S6D, S7D). Representative images and quantification of foci formation induced by the indicated cells (n=3).

(S6E, S7E) Cells were synchronized at the G2/M boundary and assessed with PI-stained flow cytometry after release. Cell cycle profiles showed the G1 cell population at 4 hours post-release (n=3).

Mean ± SD. P values by paired (B) or unpaired Student's t test (A, C-E). *P <.05, **P <.01, ***P < .001, ****P < .0001. ns: not significant.

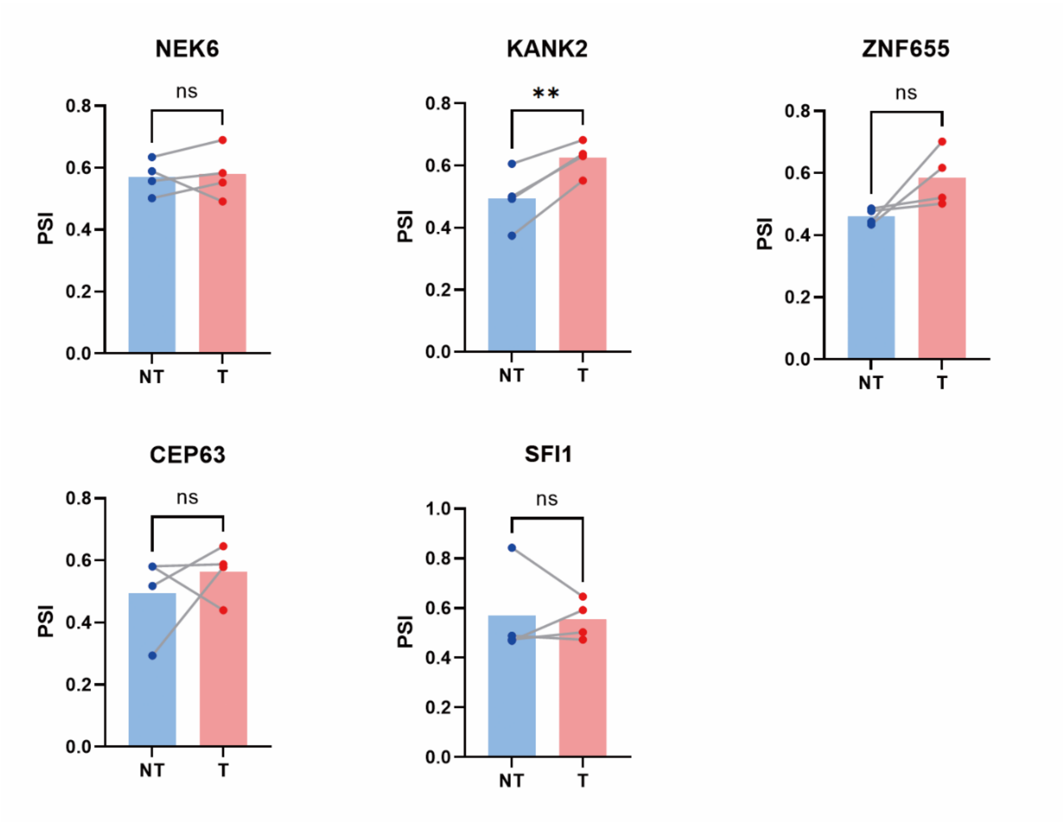

Fig. S8. The splicing efficiency of these five targets in 4 pair of clinical samples were validated using RT-PCR and agarose gel electrophoresis. The percentages of cassette exon inclusion over the total transcripts were presented using PSI values.

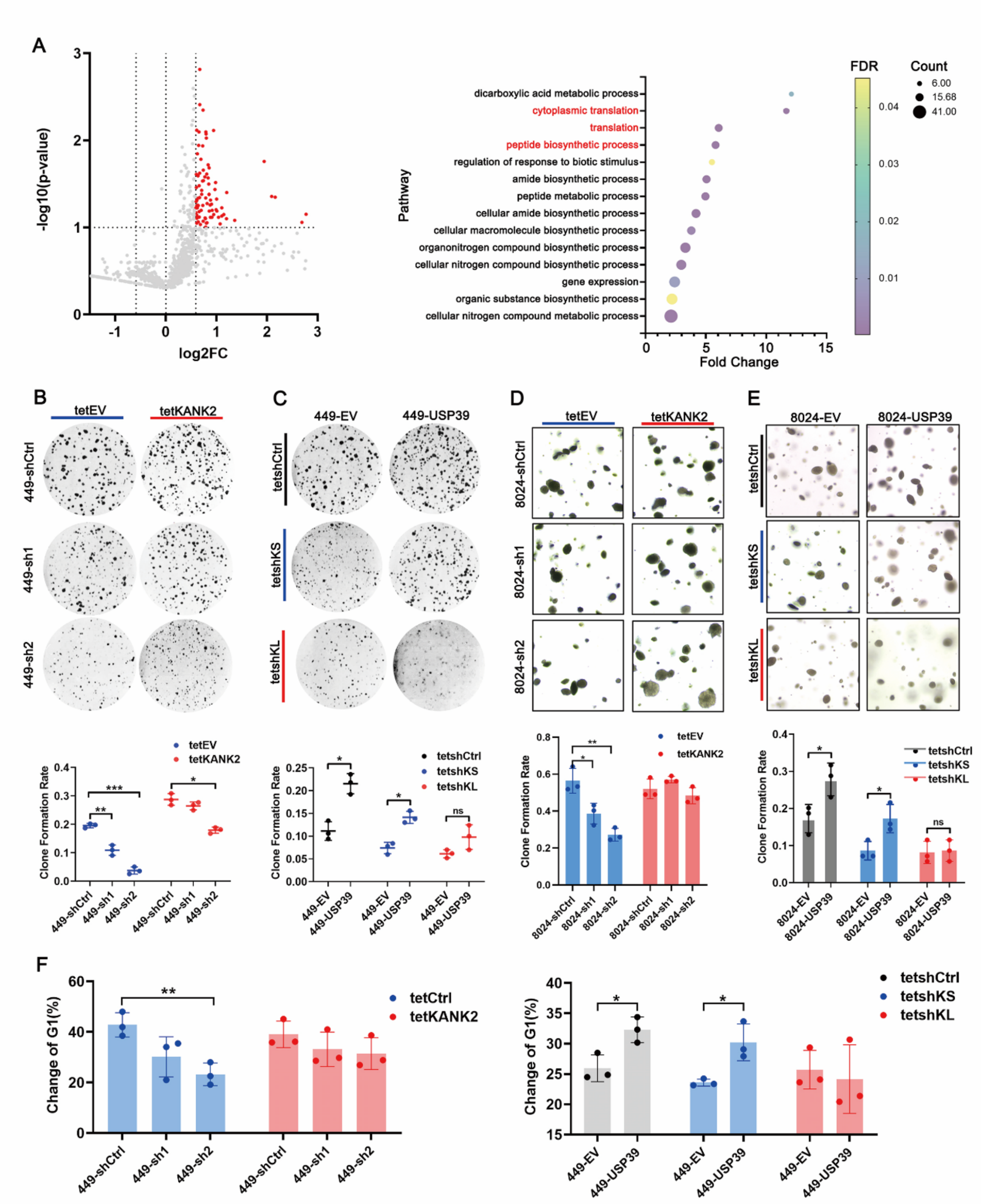

Fig. S9. KANK2-L exhibits stronger pro-proliferative activities than KANK2-S.

(A) Volcano plot was generated from RNA pulldown mass spectrometry analysis to compare KANK2 S and KANK2 L interactors: red splashes showed the genes significantly enriched in KANK2-L pulldown samples than in KANK2-

S pulldown samples (left). The GO BP enrichment analysis was performed using the above mentioned KANK2-L enriched genes (right).

(B, C) Tet-KANK2 was introduced to USP39-deficient *SNU-449* cells (B) and Tet-shKANK2-L(tetshKL) and Tet-KANK2-S (tetshKS) were introduced into USP39-overexpressing *SNU-449* cells (C). Cells were treated with 1μg/ml DOX and foci formation assay was performed to access growth of the indicated cells. Representative images and statistical results are shown (n=3).

(D, E) Tet-KANK2 was introduced to USP39-deficient *PLC-8024* cells (D) and Tet-shKANK2-L(tetshKL) and Tet-KANK2-S (tetshKS) were introduced into USP39-overexpressing *PLC-8024* cells (E). After treatment with 1μg/ml DOX, soft agar formation assay was performed to access anchorage-independent growth of the indicated cells. Representative images and statistical results are shown (n=3).

(F) Tet-KANK2 was introduced to USP39-deficient *SNU-449* cells (left) and Tet-shKANK2-L(tetshKL) and Tet-KANK2-S (tetshKS) were introduced into USP39-overexpressing *SNU-449* cells (right). After induced with 1μg/ml DOX, cells were treated with Thd and nocodazole to synchronize them at G2/M boundary. Following the release, the cells arrested in G2/M (0h) would reenter G1 phase (4h). Cell cycle profiles showed changes in G1 cell populations at 4 hours post-release (n=3).

Mean ± SD. p values by Pearson's correlation coefficient (A) and unpaired Student's t test (B-F). ∗P < .05, ∗∗P < .01, ∗∗∗P < .001. ns: not significant.

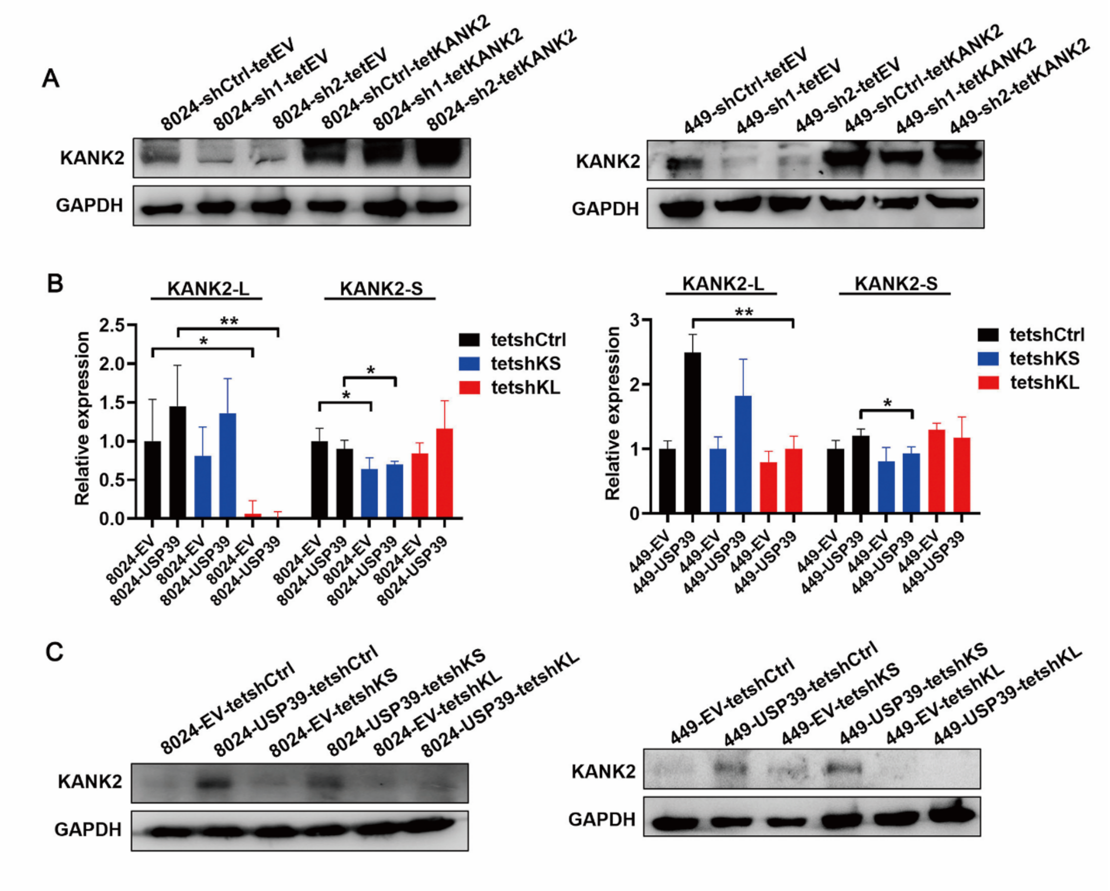

Fig. S10. Tet-On system mediates KANK2 overexpression and isoform-specific knockdown.

(A) Tet-On system mediated KANK2 overexpression in USP39-deficient *PLC-8024* and *SNU-449* cells. The overexpression effects were verified by WB.

(B, C) Tet-shKANK2-L(tetshKL) and Tet-KANK2-S (tetshKS) were introduced into USP39-overexpressing *PLC-8024* and *SNU-449* cells. The knockdown effects were examined by qRT-PCR (B) and WB (C). KANK2-L depletion reduced KANK2 protein expression, while KANK2-S targeting barely affected KANK2 protein expression despite successful knockdown of KANK2-S mRNA.

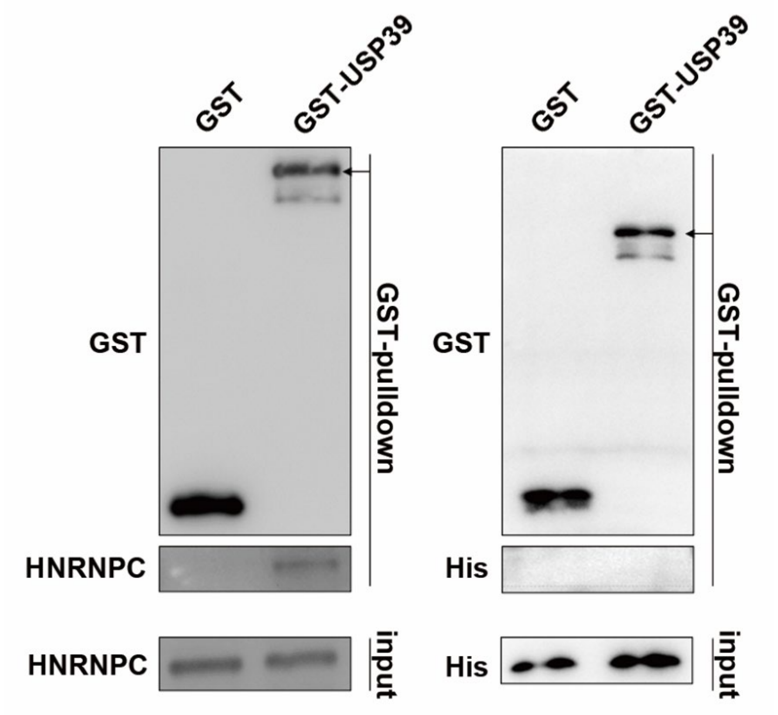

Fig. S11. The GST-pulldown assay verified the interaction between USP39 and HNRNPC (left)/SRSF6 (right).

GST-pulldown experiments with GST-USP39 and His-HNRNPC (left)/SRSF6 (right) recombinant proteins and visualized by WB analysis using anti-GST and anti-HNRNPC (left)/anti-His (right) antibodies, respectively.

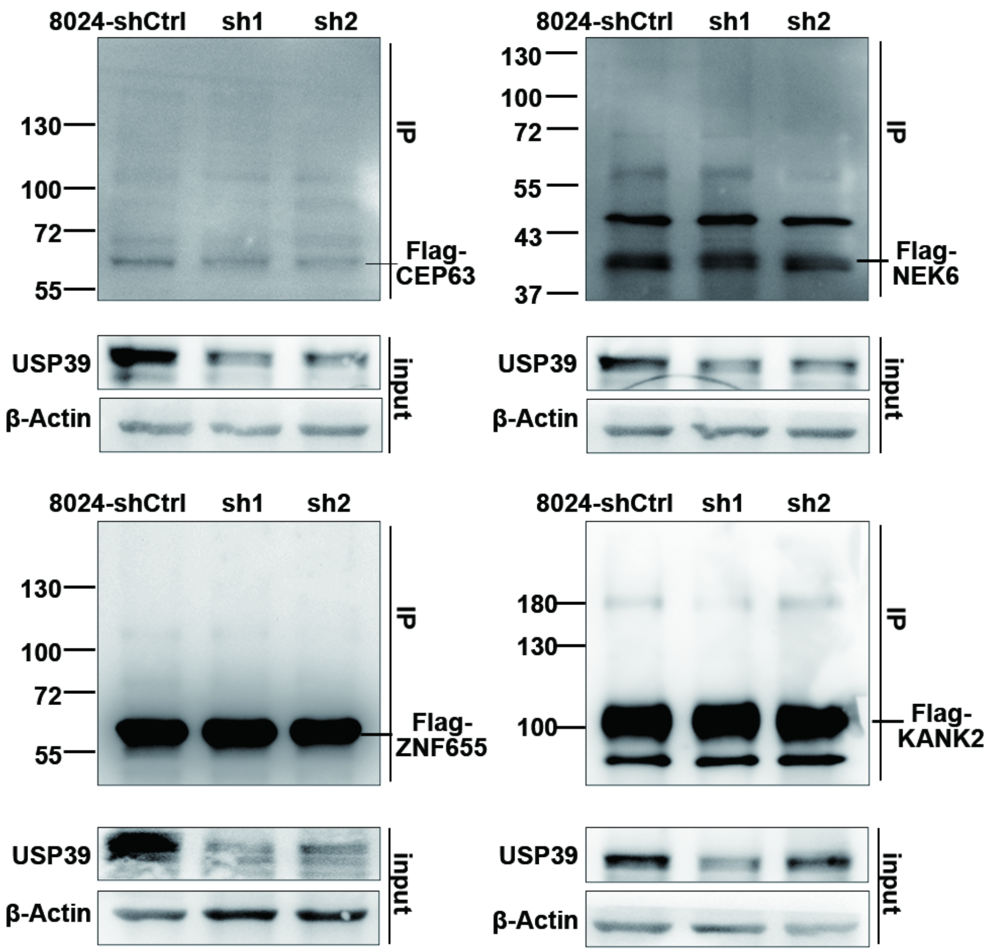

Fig. S12. Flag-CEP63, Flag-NEK, Flag-ZNF655 or Flag-KANK2 were co-transfected with HA-Ubiquitin in control and USP39-deficient *PLC-8024* cells. Immunoprecipitation assays were conducted using an antibody against Flag and visualized by WB using anti-Flag and anti-HA antibodies.
